# Blimp-1/PRDM1 and Hr3/RORβ specify the blue-sensitive photoreceptor subtype in *Drosophila* by repressing the hippo pathway 

**DOI:** 10.3389/fcell.2023.1058961

**Published:** 2023-03-07

**Authors:** Joseph Bunker, Mhamed Bashir, Sydney Bailey, Pamela Boodram, Alexis Perry, Rory Delaney, Maria Tsachaki, Simon G. Sprecher, Erik Nelson, Gerald B. Call, Jens Rister

**Affiliations:** ^1^ Department of Biology, Integrated Sciences Complex, University of Massachusetts Boston, Boston, MA, United States; ^2^ NYU Langone Medical Center, New York, NY, United States; ^3^ Department of Biology, University of Fribourg, Fribourg, Switzerland; ^4^ Arizona College of Osteopathic Medicine, Midwestern University, Glendale, AZ, United States; ^5^ Department of Pharmacology, College of Graduate Studies, Midwestern University, Glendale, AZ, United States

**Keywords:** rhodopsin, retina, photoreceptor, ecdysone, BLIMP-1, rorβ, color vision, hippo pathway

## Abstract

During terminal differentiation of the mammalian retina, transcription factors control binary cell fate decisions that generate functionally distinct subtypes of photoreceptor neurons. For instance, Otx2 and RORβ activate the expression of the transcriptional repressor Blimp-1/PRDM1 that represses bipolar interneuron fate and promotes rod photoreceptor fate. Moreover, Otx2 and Crx promote expression of the nuclear receptor Nrl that promotes rod photoreceptor fate and represses cone photoreceptor fate. Mutations in these four transcription factors cause severe eye diseases such as retinitis pigmentosa. Here, we show that a post-mitotic binary fate decision in *Drosophila* color photoreceptor subtype specification requires ecdysone signaling and involves orthologs of these transcription factors: *Drosophila* Blimp-1/PRDM1 and Hr3/RORβ promote blue-sensitive (Rh5) photoreceptor fate and repress green-sensitive (Rh6) photoreceptor fate through the transcriptional repression of *warts*/*LATS*, the nexus of the phylogenetically conserved Hippo tumor suppressor pathway. Moreover, we identify a novel interaction between Blimp-1 and *warts,* whereby Blimp-1 represses a *warts* intronic enhancer in blue-sensitive photoreceptors and thereby gives rise to specific expression of *warts* in green-sensitive photoreceptors. Together, these results reveal that conserved transcriptional regulators play key roles in terminal cell fate decisions in both the *Drosophila* and the mammalian retina, and the mechanistic insights further deepen our understanding of how Hippo pathway signaling is repurposed to control photoreceptor fates for *Drosophila* color vision.

## 1 Introduction

**FIGURE 1:**
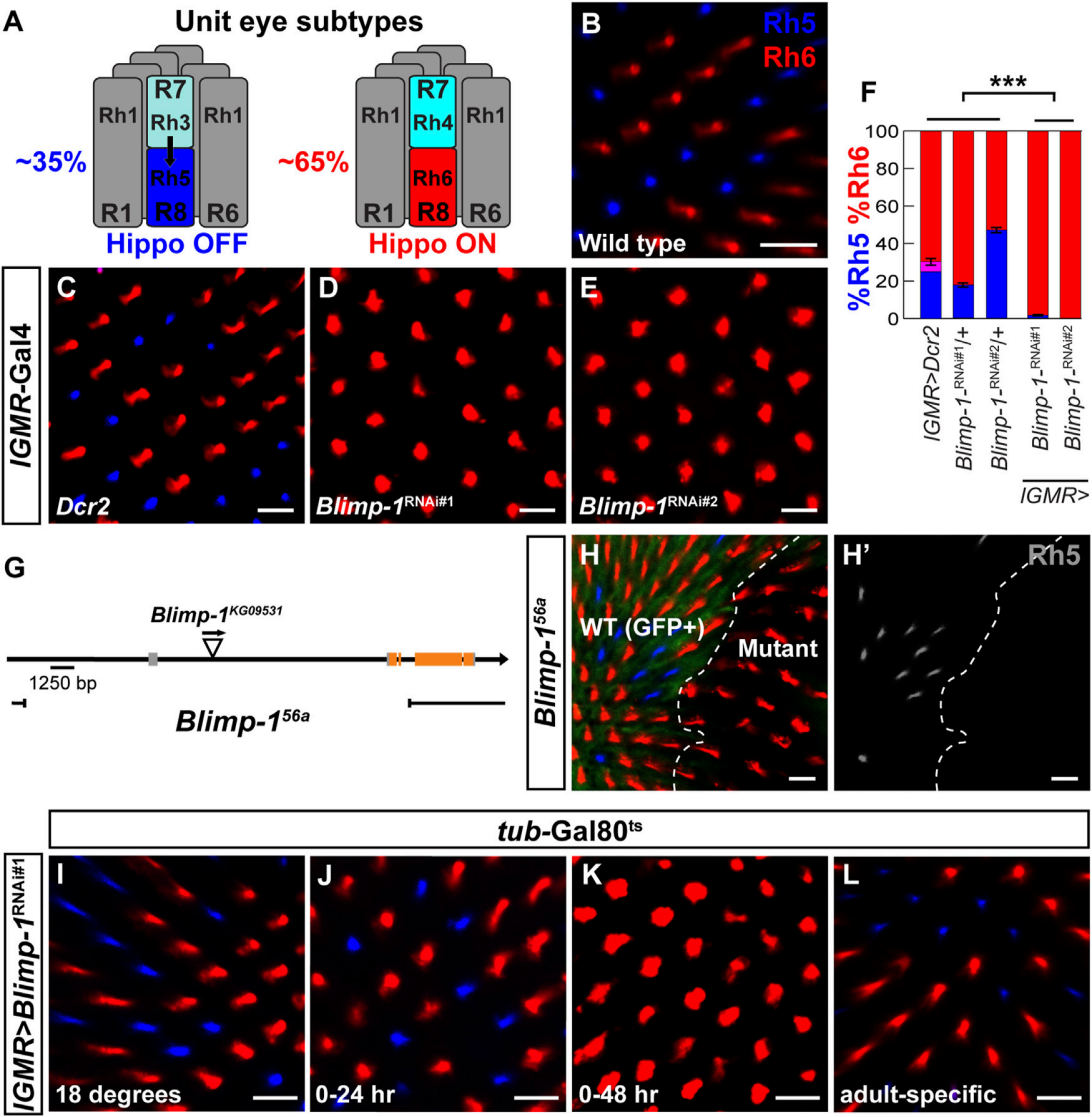
Blimp-1 is required to promote Rh5 and to repress Rh6. **(A)** Schematic side view of the two different unit eye subtypes in the *Drosophila* retina. Black arrow represents the Activin signal from Rh3-expressing R7 photoreceptors (35%, left) that induces Rh5 fate (Hippo OFF) in the proximally located R8 photoreceptors. Rh4-expressing R7 photoreceptors (65%, right) do not send the Activin signal and the corresponding R8 photoreceptors express Rh6 (Hippo ON) by default. **(B–E)** Confocal images of whole mounted adult retinas stained with antibodies for Rh5 (blue) and Rh6 (red). **(B)** A wild-type Canton S retina has an Rh5:Rh6 ratio of ∼35:65. **(C)** Expression of *Dcr2* with the pan-photoreceptor driver *lGMR-Gal4* does not affect the Rh5:Rh6 ratio. **(D and E)** Expression of two independent *Blimp-1* RNAi constructs, *Blimp-1-*RNAi^kk^ (*Blimp-1* ^*RNAi#1*^) and *Blimp-1-*RNAi^TRiP #36634^ (*Blimp-1* ^*RNAi#2*^), in combination with *Dcr2* causes a dramatic loss of Rh5 and a corresponding gain of Rh6. **(F)** Quantification of R8 subtypes in controls and *Blimp-1* knockdowns. Graph shows the percentage of R8 photoreceptors that express exclusively Rh5 (blue), exclusively Rh6 (red), or co-express Rh5 and Rh6 (magenta). Mean %Rh6 was compared among genotypes with an ANOVA and a *post hoc* Tukey HSD Test; ***p < 0.0001. 7-8 retinas were scored for each genotype. **(G)** Schematic showing the location of the *Blimp-1* ^*56a*^ deletion that was generated through imprecise P-element (triangle) excision. **(H)** Confocal image showing wild type R8 photoreceptors (GFP positive, left) exhibiting a normal Rh5:Rh6 ratio and *Blimp-1* ^*56a*^ null mutant clones (GFP negative, right) that exclusively express Rh6. The white dashed line denotes the boundary between wild-type and *Blimp-1* mutant tissue. **(H′)** Rh5 channel (grayscale) shows the loss of Rh5 in the *Blimp-1* mutant tissue. **(I–L)** Confocal images of temporally restricted *lGMR > Blimp-1* ^*RNAi#1*^ knockdown with *tub-*Gal80^ts^. **(I)** Control flies raised throughout development and adulthood at 18°C, i.e., no *Blimp-1* knockdown, have a normal Rh5:Rh6 ratio. **(J)** Pupal *Blimp-1* knockdown from 0 to 24 h after puparium formation at 29°C does not affect the Rh5:Rh6 ratio. **(K)** Pupal *Blimp-1* knockdown from 0 to 48 h after puparium formation at 29°C causes a loss of Rh5 and gain of Rh6. **(L)** Adult-specific *Blimp-1* knockdown for 7 days at 29°C following eclosion does not affect the Rh5:Rh6 ratio. All scale bars, 10 µm.

Color vision requires the expression of light-sensing pigments with different wavelength-sensitivities in different photoreceptor (PR) subtypes (Rister and Desplan, 2011). For instance, human color vision is based on three cone PR subtypes that express short-, medium-, or long wavelength-sensitive pigments (Nathans, 1999; Hofer et al., 2005). Likewise, the *Drosophila melanogaster* retina expresses five color-sensing Rhodopsin (Rh) pigments in distinct PR subtypes (Poupault et al., 2021). The “outer” PRs R1-R6 express blue/green-sensitive Rh1 and mediate dim light vision equivalent to human rods (O'Tousa et al., 1985; Zuker et al., 1985). The two ‘inner’ PRs R7/R8, which are arranged in tandem, each occur in two subtypes (Figure 1A) that are sensitive to different wavelengths and mediate color vision equivalent to human cones (Roorda and Williams, 1999). 35% of R7 PRs express the short UV-sensitive Rh3 and are coupled to proximally located R8 PRs that express the blue-sensitive Rh5 (Figure 1A, left), while the other 65% of R7 PRs express the long UV-sensitive Rh4 coupled with R8 PRs that express the green-sensitive Rh6 (Figure 1A, right) (Fortini and Rubin, 1990; Chou et al., 1996; Papatsenko et al., 1997; Chou et al., 1999).

The binary cell-fate decision to express either Rh5 or Rh6 in R8 PRs (Figure 1B) requires an Activin/TGFβ signal from the distally located R7 PRs (arrow in Figure 1A) (Wells et al., 2017) and differential regulation of the Hippo tumor suppressor pathway in R8 PRs (Mikeladze-Dvali et al., 2005; Jukam and Desplan, 2011; Jukam et al., 2013). In the canonical and conserved role of the Hippo pathway (Zeng and Hong, 2008; Zhao et al., 2008; Zhang et al., 2009; Chan et al., 2011; Halder and Johnson, 2011; Harvey and Hariharan, 2012; Halder and Camargo, 2013), the kinase Warts (Wts) restricts tissue growth by phosphorylating the transcriptional co-activator and oncogene Yorkie (Yki), which prevents Yki from entering the nucleus (Huang et al., 2005; Oh and Irvine, 2008). When Yki is not phosphorylated, it enters the nucleus and associates with transcription factors such as Scalloped (Sd) to activate genes that function in promoting growth, inhibiting apoptosis, and negative feedback regulation (Hamaratoglu et al., 2006; Neto-Silva et al., 2010).

**FIGURE 2:**
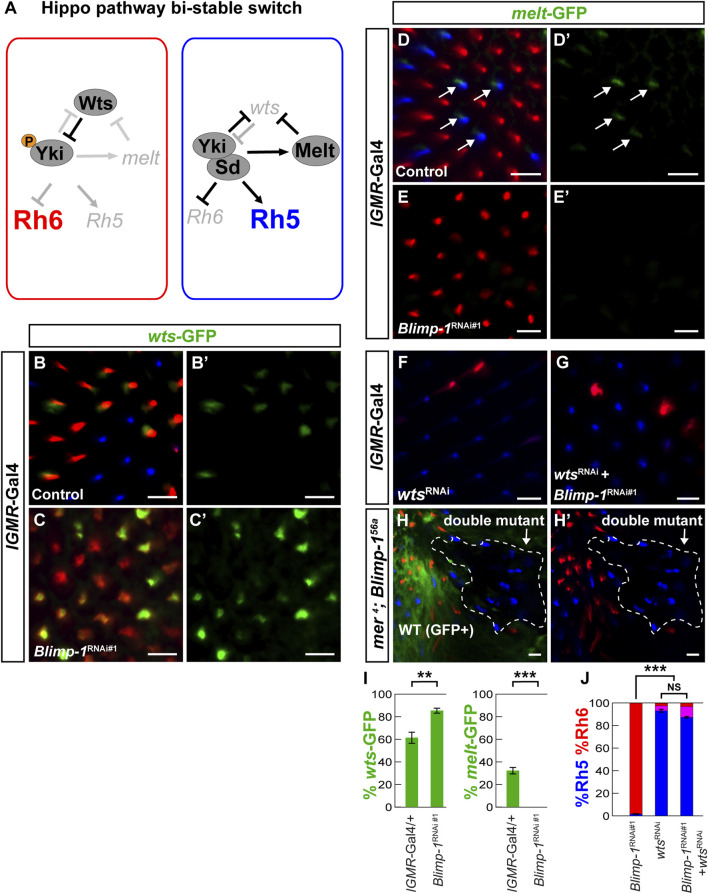
Blimp-1 is required for *warts* repression and *melted* expression. **(A)** Schematic of the repurposed Hippo pathway that controls a binary Rh5 vs. Rh6 cell fate decision in post-mitotic R8 photoreceptors. In Rh6 expressing photoreceptors (left), Wts represses Yki by phosphorylation, which prevents the expression of *melt* and *Rh5* and permits Rh6 expression. In Rh5 expressing photoreceptors (right), Yki binds to Sd, activates *melt* and *Rh5*, and represses *wts* and *Rh6.* **(B)** In the *lGMR-Gal4*/+ driver control, the *wts-*GFP reporter is expressed in most Rh6 photoreceptors and absent in all Rh5 photoreceptors. **(C)** In *lGMR > Blimp-1* ^*RNAi#1*^ retinas, *wts-*GFP is de-repressed with Rh6. **(B′,C′)** GFP channel. **(D)** In the *lGMR-Gal4*/+ driver control, the *melt-*GFP reporter is expressed in most Rh5 photoreceptors but absent in Rh6 photoreceptors. White arrows indicate *melt-*GFP expression. **(E)** In *lGMR > Blimp-1* ^*RNAi#1*^ retinas, *melt-*GFP expression is completely lost. **(D′, E′)** GFP channels. **(F)** Knockdown of *wts* with *lGMR-Gal4* causes a gain of Rh5 and loss of Rh6. **(G)** Knockdown of *Blimp-1* in addition to *wts* does not modify the *wts* knockdown phenotype. **(H)** *mer* ^*4*^; *Blimp-1* ^*56a*^ double mutant clones (encircled by the white dashed line) exclusively express Rh5, which is the opposite of *Blimp-1* ^*56a*^ mutant clones that exclusively express Rh6. All scale bars, 10 µm. **(I)** Quantification of the *wts-*GFP-expressing R8 photoreceptors (left graph) and the *melt-*GFP expressing R8 photoreceptors (right graph) in the driver control and *Blimp-1* knockdown. Mean %*wts-*GFP (left) or %*melt-*GFP reporter (right) expression in driver control vs. knockdown was compared with an ANOVA and a *post hoc* Tukey HSD Test; ***p < 0.0001. 6 retinas were analyzed for each genotype. **(J)** Quantification of R8 subtypes, mean %Rh5 was compared among genotypes with an ANOVA and a *post hoc* Tukey HSD Test; ***p < 0.0001.5-8 retinas were scored for each genotype.

In terminally differentiating *Drosophila* PRs, the Hippo pathway is repurposed to promote green-sensitive Rh6 PR fate, while its inactivation promotes blue-sensitive Rh5 PR fate (Figure 2A) (Mikeladze-Dvali et al., 2005; Jukam and Desplan, 2011; Jukam et al., 2013; Pojer et al., 2021). In this post-mitotic context, Yki/Sd promote Rh5 fate and inactivate the Hippo pathway by repressing *wts* at the transcriptional level (Mikeladze-Dvali et al., 2005; Jukam et al., 2013; Xie et al., 2019). *Wts* repression results in the activation of *melted* (*melt*), which encodes a Pleckstrin Homology domain protein (Mikeladze-Dvali et al., 2005; Teleman et al., 2005). For *wts* repression and *melt* activation, Yki/Sd require the transcription factors Orthodenticle (Otd) and Traffic Jam (Tj), which act in a coherent feedforward loop (Vandendries et al., 1996; Mears et al., 2001; Tahayato et al., 2003; Montana et al., 2011; Fichelson et al., 2012; Hao et al., 2012; Jukam et al., 2013)*.* Conversely, when *wts* is expressed, it represses Yki; this leads to the transcriptional repression of *melt*, which allows the Hippo pathway to remain active and to promote Rh6 fate (Figure 2A). Thus, the Hippo pathway functions as a bi-stable switch in post-mitotic PRs: *wts* expression (Hippo ON) and *melt* repression specify Rh6 fate, while *melt* expression and *wts* repression (Hippo OFF) specify Rh5 fate (Figure 2A). The transcriptional regulation of *wts* in this context remains poorly understood.

Here, we show that *Drosophila* color PR subtype specification requires ecdysone signaling as well as the orthologs of transcription factors that promote rod PR fate in the mammalian retina: B lymphocyte-induced maturation protein-1/PR domain containing 1 (Blimp-1/PRDM1) and Hr3 (RAR-related orphan receptor β/RORβ) repress the Hippo pathway in post-mitotic *Drosophila* PRs, thereby promoting blue-sensitive Rh5 PR fate and repressing green-sensitive Rh6 PR fate. Moreover, we identify a novel mechanism of *wts* regulation in which Blimp-1 represses a *wts* enhancer to give rise to Rh6 PR subtype-specific *wts* expression. However, we find that this regulation of the Hippo pathway by Blimp-1 is context-specific, as Blimp-1 is required for wing growth independently of Wts. In summary, these results show that conserved transcriptional regulators play key roles in terminal PR differentiation in both *Drosophila* and mammals. Moreover, these insights deepen our understanding of the mechanisms that allow the Hippo pathway to control a binary cell-fate decision in terminally differentiating color PRs.

## 2 Materials and methods

### 2.1 Stocks and maintenance

The following *Drosophila* stocks were used in this study: *mer* ^*4*^ *FRT19A* (Fehon et al., 1997), *ey-FLP, P{ubiGFP} FRT19A* (from Jessica Treisman, NYU), *ey-FLP, P{ubiGFP} FRT80* (from Claude Desplan, NYU), *lGMR-Gal4* (Wernet et al., 2006), UAS-Babo* (Wells et al., 2017), *sens-Gal4* (from Graeme Mardon, Baylor College of Medicine), and *ex-LacZ* (Yu and Pan, 2018). From Bloomington *Drosophila* Stock Center, we obtained: *MS1096-Gal4; UAS-Dcr2* (#25706), *UAS-Dcr2; nubbin-Gal4* (#25754), *tubP-Gal80* ^*ts*^ (#7017), *en2.4-Gal4* (#30564), *UAS-Dcr2; en2.4-Gal4, UAS-2XEGFP* (#25752), *UAS-Hr3-RNAi* #1 (TRiP.JF02542, #27253), *UAS-Hr3-RNAi* #2 (TRiP.JF02543, #27254), *UAS-wts-RNAi* (TRiP HMS00026) (#34064), *UAS-EcR-RNAi* (TRiP.HMJ22371, #58286), *UAS-EcR-A-dsRNA* (#9328), *UAS-EcR-b1-dsRNA* (#9329), *UAS-Blimp-1-RNAi* #2 (TRiP.GL00594, #36634), and *Blimp-1* ^*KG09531*^ (#15195). From Vienna *Drosophila* Resource Center, we obtained *UAS*-*Blimp-1-RNAi* #1 (KK #108374).

All stocks were maintained on standard lab food at 25°C, 50% humidity, and under a 12 h/12 h light/dark cycle. All experiments and controls were conducted at 25°C with 2–5 day-old female flies, unless stated otherwise. For RNAi experiments with the *sens*-*Gal4* driver, third instar larvae of the experimental and control crosses were shifted from a 25°C incubator to a 29°C incubator.

For staging of pupae, newly formed white prepupae—corresponding to 0 h after puparium formation—were circled with a marker pen on the food vial (Hsiao et al., 2012). At the desired time point after puparium formation, pupae were gently removed from the vial using forceps. For Gal80^ts^ experiments, white prepupae were shifted from a 25°C incubator to a 29°C incubator and then shifted at the desired time point (see Results) from the 29°C incubator to an 18°C incubator.

### 2.2 Generation of the *Blimp-1* null mutant

We used the P-element insertion *Blimp-1* ^*KG09531*^ to generate the imprecise excision allele *Blimp-1* ^*56a*^, following standard procedures (Engels et al., 1990). The deletion breakpoints of the imprecise excision allele were initially determined by Next-Generation Sequencing of the 3L chromosome from predominantly homozygous *Blimp-1* ^*56a*^ mutant first instar larvae that had reduced or no GFP expression from the *TM3, P{GAL4-Kr.C}DC2*, *P{UAS-GFP.S65T}DC10*, *Sb* ^*1*^ balancer chromosome. Initial breakpoints were determined by using the bioinformatics tool BreakDancer (Fan et al., 2014) to evaluate the chromosome for large structural variants. BreakDancer predicted a deletion from bases 3L:5624169.5645326, lining up with an area of low sequence coverage noticeable in the alignment. Subsequent Sanger sequencing surrounding this region—using the forward primer TTT​TCC​AGG​TCA​TCG​TTT​CC and the reverse primer ATCGTCGTCTCAGGATCCAC—revealed that the chromosomal region that was deleted is 3L:5624036.5645326 (Figure 1G), based off the *D. melanogaster* genome release 6.45.

### 2.3 Generation of *warts* and *melted* reporters

The first *melt* intron was digested with KpnI and NotI to obtain a 2 kb enhancer fragment from its 3′ region (Jukam et al., 2013); the second *wts* intron (in TOPO plasmid/ThermoFisher, a gift from David Jukam) was digested with BamHI, BglII, and XhoI to obtain a 2.6 kb enhancer fragment from its 3’ region. The obtained enhancer sequences were confirmed by Sanger sequencing. The following primers were used to mutate the two Blimp-1 motifs in the *wts* intron (mutated bases are in bold) with the Q5^®^ Site-Directed Mutagenesis Kit (New England Biolabs).

*wts-*ΔBlimp-1 motif 1 forward: **aca**cgctAATCCACTTAAGCTCCTCTG
*wts-*ΔBlimp-1 motif 1 reverse: **a**tg**cca**cGG​CAA​TTC​TAC​GGT​TCG​TG
*wts-*ΔBlimp-1 motif 2 forward: a**c**gcC​AAT​GGG​CTC​ACT​AAA​TC
*wts-*ΔBlimp-1 motif 2 reverse: t**a**gc**a**AAT​TTG​CCG​TAA​ATT​GGC

We inserted the wild type or mutant enhancer constructs into a transformation plasmid containing an *egfp* reporter gene, a *mini-white* ^*+*^ transformation marker, an *hsp70* minimal promoter, and an *attB* site for *phiC31*-mediated transgenesis (Rister et al., 2015). The constructs were inserted in the second chromosomal landing site attP40 or the third chromosomal landing site J36 (ZH-attP-86Fb) (Bischof et al., 2007).

### 2.4 Luciferase reporter assay

The *wts-hsp70* enhancer-promoter construct (see above) was amplified using the Phusion^®^ High-Fidelity PCR Kit (New England Biolabs) and fused to a firefly *luciferase* reporter gene (*wts-hsp70-luc*), digested with BglII and KpnI, and subcloned into the pGL3-basic vector (Promega) with the NEBuilder^®^ HiFi DNA Assembly master mix (New England Biolabs). The following primers were used to amplify *wts-hsp70* (pGL3 overhangs are in lowercase).

*wts-hsp70* forward: att​tct​cta​tcg​ata​ggt​acC​TGA​CAT​ATT​TGG​TGC​TAC​ACA​TGT​AAT​CCC
*wts-hsp70* reverse: cca​agc​tta​ctt​aga​tcg​caG​GTG​GCG​ACC​GGT​GGA​TC

To generate the *Blimp-1* expression construct (*Ac5-Blimp-1*), *Blimp-1-RA* cDNA was amplified from a *UAS-Blimp-1-RA* plasmid (*Drosophila* Genomics Resource Center #1634032) and inserted into the pAc5.1/V5-His A vector (Invitrogen) by digest with XhoI and AgeI and using the NEBuilder^®^ HiFi DNA Assembly master mix. The following primers were used to amplify the *Blimp-1-RA* insert (pAc5.1/V5-His overhangs are in lowercase).

*Blimp-1RA* forward: tcc​agc​aca​gtg​gcg​gcc​gcA​GTT​TCC​CGT​AAG​CAA​CAA​AAC
*Blimp-1RA* reverse: aat​ggt​gat​ggt​gat​gat​gat​ACG​TGC​ATT​CGA​TGA​TCA​TG

To generate the *Hr3* expression construct (*Ac5-Hr3*), we ordered the *Hr3-RA* coding sequence inserted into the p-UCIDT-AMP vector from Integrated DNA Technologies (IDT). From that plasmid we amplified *Hr3-RA* and inserted it into the pAc5.1/V5-His A vector (Invitrogen) by digest with NotI and EcoRI and using the NEBuilder^®^ HiFi DNA Assembly master mix. The following primers were used to amplify the *Hr3-RA* insert (pAc5.1/V5-His overhangs are in lowercase).

*Hr3-1RA* forward: cta​cta​gtc​cag​tgt​ggt​ggA​TGT​ATA​CGC​AAC​GTA​TGT​TTG
*Hr3-1RA* reverse: agg​gcc​ctc​tag​act​cga​gcT​TAT​GTC​AGG​TCC​TGC​TG

Luciferase reporter assays were performed using the Dual-Luciferase^®^ Reporter Assay System (Promega). For all experiments and controls, 100 ng of *wts-hsp70-luc* were co-transfected with 100 ng of a *Renilla luciferase* gene fused to a *TK* promoter (*pRL-TK*) (Promega) that served as a control reporter. For controls, *wts-hsp70-luc* and *pRL-TK* were co-transfected with 100 ng of pAc5.1/V5-His A empty vector; for experiments, *wts-hsp70-luc* and *pRL-TK* were co-transfected with 100 ng of either *Ac5-Blimp-1* or *Ac5-Hr3. Drosophila* S2 cells (Gibco) were maintained in Schneider’s Medium with 10% fetal bovine serum (Gibco) at room temperature. 1 × 10^6 cells were plated in 6-well tissue culture dishes (Corning) 24 h prior to transfection with 7.5 μL Effectene Transfection Reagent (Qiagen). Samples were transfected in triplicate for each experiment, and each experiment was performed at least three independent times. Background luminescence was determined using non-transfected cells and subtracted from control and experimental luminescence readings. Firefly luminescence results were normalized to *Renilla* luminescence results and presented as Relative Luminescence Units (RLU). Error bars for luciferase data represent ±S.E.M.

### 2.5 Wing size assay

Adult wings of female flies raised at 25°C were removed with forceps and mounted with mounting medium–lactic acid/CMCP-10 high viscosity mountant (Polysciences) (1:3, v/v)—on a glass slide. 5 μL of isopropanol (Sigma-Aldrich) was added to the wings and allowed to evaporate without drying out the wings. Next, 10 µL of mounting medium was added to the wings on the slide, wings were oriented, and air bubbles were removed. To determine relative wing sizes, wing areas were calculated using ImageJ. At least five wings were measured per genotype, and average wing areas were normalized to the average wing area of the *MS1096-Gal4*/+ driver control. Error bars indicate the standard error of the mean (S.E.M.).

### 2.6 Imaging the *Drosophila* eye

Adult flies were embedded in an agarose gel that was prepared in a 500 mL Erlenmeyer flask by mixing 2 g of UltraPure Agarose (Invitrogen) with 100 mL of distilled water. The mixture was microwaved until bubbles were seen and the agarose was fully dissolved. Next, the Erlenmeyer flask with the dissolved agarose was transferred to a 60°C water bath (Thermo Scientific) to cool the agarose gel but maintaining it liquid. Flies were anesthetized with CO_2_ and transferred to a 60 mm Petri dish (Falcon) filled approximately halfway with the liquid agarose gel. Wings and legs were submerged and the head was oriented with forceps such that one compound eye faced the microscope lens. Next, the Petri dish was placed on ice to allow the gel to solidify and then positioned under a Stemi 508 Trinoc microscope (model #4350649030, Zeiss); the eye was imaged using an Axiocam 208 HD/4k color camera (model #4265709000) set to auto exposure and auto white balance. Image processing was performed using Fiji (https://imagej.net/software/fiji/), Adobe Photoshop 2020, and Adobe Illustrator 2020 software.

### 2.7 Retina whole-mounts, immunohistochemistry, and confocal microscopy

We performed adult retina dissections and immunohistochemistry as previously described (Hsiao et al., 2012). We dissected adult retinas in cold phosphate-buffered saline (PBS) and fixed them in 3.7% formaldehyde solution (in PBS) for 15 min at room temperature. After two washes with PBS and one with PBST (PBS +0.2% Triton-X, Sigma), we removed the laminas. Next, we incubated the retinas overnight in the primary antibodies that were diluted in PBST (sheep anti-GFP—from AbD Serotec—1:100; mouse anti-Rh3—from Steve Britt/University of Texas—1:10; mouse anti-Rh5—from Steve Britt—1:400, guinea pig anti-Rh4—from Claude Desplan/NYU—1:1,000; or rabbit anti-Rh6—from Claude Desplan—1:1,000). After three PBST washes, we incubated the retinas overnight at room temperature in the secondary antibodies (Alexa Fluor 488-, 555-, or 647-conjugated raised in donkey; Molecular Probes) that were diluted 1:800 in PBST. Alexa Fluor (488 or 555)-coupled Phalloidin (1:150, Invitrogen) was used to visualize the photoreceptor rhabdomeres. After three PBST washes, we mounted the retinas on bridge slides with SlowFade (Molecular Probes).

We performed pupal retina dissections as previously described but with some modifications (Hsiao et al., 2012; Wang et al., 2022). We circled white prepupae, which were raised at 25°C, for staged pupal dissections. At the desired stage (after 24, 48, or 72 h, respectively), pupae were taped to a dissecting plate with double-sided tape and removed from the pupal case with forceps. The pupa was then submerged in ice-cold PBS and the head was removed using microdissection scissors. Next, the retina-brain complexes were removed using forceps and fixed in 3.7% formaldehyde solution (in PBS) for 15 min at room temperature. After two washes with PBS and one wash with PBST, the retina-brain complexes were incubated at 4°C overnight in primary antibodies (guinea pig anti-Blimp-1—from Sudipto Roy/National University of Singapore—1:400; rat anti-Elav—from DHSB—1:50) diluted in PBST and 5% normal donkey serum. After three PBST washes, we incubated the retinas overnight at 4°C for three hours in the secondary antibodies (see above). After three PBST washes, we performed a secondary fixation step by submerging retina-brain complexes in 3.7% formaldehyde solution (in PBS) for 20 min at room temperature. After washing three times with PBST, we removed the retina from the brain in PBS and mounted the retina on a slide using Slowfade. We imaged both adult and pupal retina whole mounts with a Leica SP5 or a Zeiss LSM 8 confocal microscope. We processed the confocal images with Fiji, Adobe Photoshop 2020, and Adobe Illustrator 2020 software.

### 2.8 Quantification of rhodopsin and reporter expression patterns

As previously described (Poupault et al., 2021), we manually scored the number of rhabdomeres that expressed the markers Rh5, Rh6, or a GFP reporter with the count tool in Adobe Photoshop 2020. The percentage of R8 PRs that expressed the respective marker was calculated for each retina as well as the mean percentage of all retinas within a genotype. Statistical comparisons across genotypes were performed using the Mann-Whitney *U* Test; significance levels are given as *p* values and error bars indicate the standard error of the mean (S.E.M.).

### 2.9 Conservation analysis of Blimp-1 motifs

To analyze the evolutionary conservation of Blimp-1 motifs, we obtained alignments of the *wts* second intron sequences from ten *Drosophila* species (Clark et al., 2007) from the UCSC genome browser (https://genome.ucsc.edu/).

## 3 Results

### 3.1 Blimp-1 is required for blue-sensitive photoreceptor subtype specification

We performed a candidate RNAi screen to identify sequence-specific transcription factors that are required for the wild type 35/65 ratio of the Rh5/Rh6-expressing R8 PR subtypes and found that the knockdown of *B lymphocyte-induced maturation protein-1* (*Blimp-1*) with the pan-PR driver *lGMR-Gal4* (Wernet et al., 2006) caused a dramatic gain of Rh6-expressing PRs and a loss of Rh5-expressing PRs (Figures 1C–F). This loss of blue-sensitive PR fate and gain of green-sensitive PR fate was observed with two independent *Blimp-1*-RNAi constructs that targeted different parts of the *Blimp-1* transcript (Figures 1D,E). To validate the RNAi results, we generated a *Blimp-1* null mutant through imprecise P-element excision (see Materials and methods), which resulted in a genomic deletion that spans from 6.8 kb upstream of the *Blimp-1* transcription start site to the third coding exon (*Blimp-1* ^*56a*^, Figure 1G). Since the *Blimp-1* ^*56a*^ null allele was embryonic lethal, we used *eyeless >* Flp to generate mutant clones in the eye with the FLP/FRT recombination system (Newsome et al., 2000). Consistent with the RNAi results, *Blimp-1* ^*56a*^ null mutant clones exclusively expressed Rh6 at the expense of Rh5, confirming that Blimp-1 is required for expression of Rh5 and repression of Rh6 (Figures 1H,H’). Taken together, Blimp-1 is required for the terminal differentiation of the blue-sensitive, Rh5-expressing R8 PR subtype.

### 3.2 Blimp-1 promotes blue-sensitive photoreceptor subtype fate in mid-pupal photoreceptors

Our next goal was to determine the time window during which Blimp-1 affects R8 PR subtype specification. We detected weak Blimp-1 expression (Ng et al., 2006) in all PRs at 24 h after puparium formation (APF) and stronger expression at 48 h APF (Supplementary Figures S1A, A’, B, B’), but no expression at 72 h APF and in adult eyes (Supplementary Figures S1C, C′, D, D′). Moreover, RNAi-mediated knockdown of *Blimp-1* abolished Blimp-1 expression (Figures S1E–F′). This suggests that Blimp-1 expression is transiently increased in PRs from early-to mid-pupal development, when R8 PR subtypes are distinguished (Jukam and Desplan, 2011), and is turned off in late pupal development. Interestingly, most of the Blimp-1 signal was not restricted to the nucleus, but surrounded it: at 48 h APF, we observed a partial overlap of Blimp-1 with the Elav nuclear marker in wild-type and occasional strong Blimp-1 PR nuclear localization in the driver control, suggesting that Blimp-1’s nuclear localization is regulated and transient. We therefore propose that Blimp-1’s nuclear entry and/or export is regulated to restrict its activity during pupal development.

Since Blimp-1 expression and nuclear localization is lost after 48 h APF, we hypothesized that Blimp-1 is required for the specification of Rh5 fate but not for its maintenance at later stages. To further investigate this transient requirement of Blimp-1 for R8 subtype specification, we used a temperature sensitive mutant of the Gal4 inhibitor Gal80 (Gal80^ts^) to temporally restrict the *Blimp-1* knockdown (McGuire et al., 2004). Permissive temperature (Figure 1I), RNAi-mediated *Blimp-1* knockdown from 0 to 24 h APF (Figure 1J), or *Blimp-1* knockdown for seven days after eclosion (Figure 1L) did not affect the Rh5:Rh6 ratio. In contrast, *Blimp-1* knockdown from 0 to 48 h APF (Figure 1K) caused a dramatic loss of Rh5 and gain of Rh6. Taken together, these data suggest that Blimp-1 is required in early to mid-pupal PRs for the specification of Rh5 fate.

We next asked if Blimp-1 acts cell autonomously in R8 PRs to promote Rh5 fate. To this end, we performed an RNAi-mediated knockdown of *Blimp-1* using the R8 driver *sens*-*Gal4* and two copies of *UAS*-*Blimp-1*-RNAi. This again led to a dramatic gain of Rh6 and loss of Rh5 (Supplementary Figures S1G–J), suggesting that Blimp-1 is required cell autonomously in R8 PRs to promote Rh5 PR fate and to repress Rh6 PR fate. In a complementary approach, we took advantage of the fact that the specification of Rh5 fate requires an Activin signal from a subset of R7 PRs (Figure 1A) that activates the type I receptor Baboon (Babo) in the proximally located subset of R8 PRs (Wells et al., 2017). If Blimp-1 acts cell autonomously in R8 PRs to promote Rh5 fate, then Blimp-1 should act downstream of the Activin signal. Indeed, when we expressed a constitutively active form of Babo (Babo*) (Wells et al., 2017) in combination with an RNAi-mediated knockdown of *Blimp-1,* Babo activation was no longer able to specify Rh5 fate and we observed the *Blimp-1* mutant phenotype (loss of Rh5 and gain of Rh6; Supplementary Figures S1K, L). In summary, these data suggest that Blimp-1 specifies Rh5 fate cell autonomously in early to mid-pupal R8 PRs downstream of the Activin signal and the Babo receptor.

### 3.3 Blimp-1 is required for the activation of *melted* and the repression of *warts*

**FIGURE 3:**
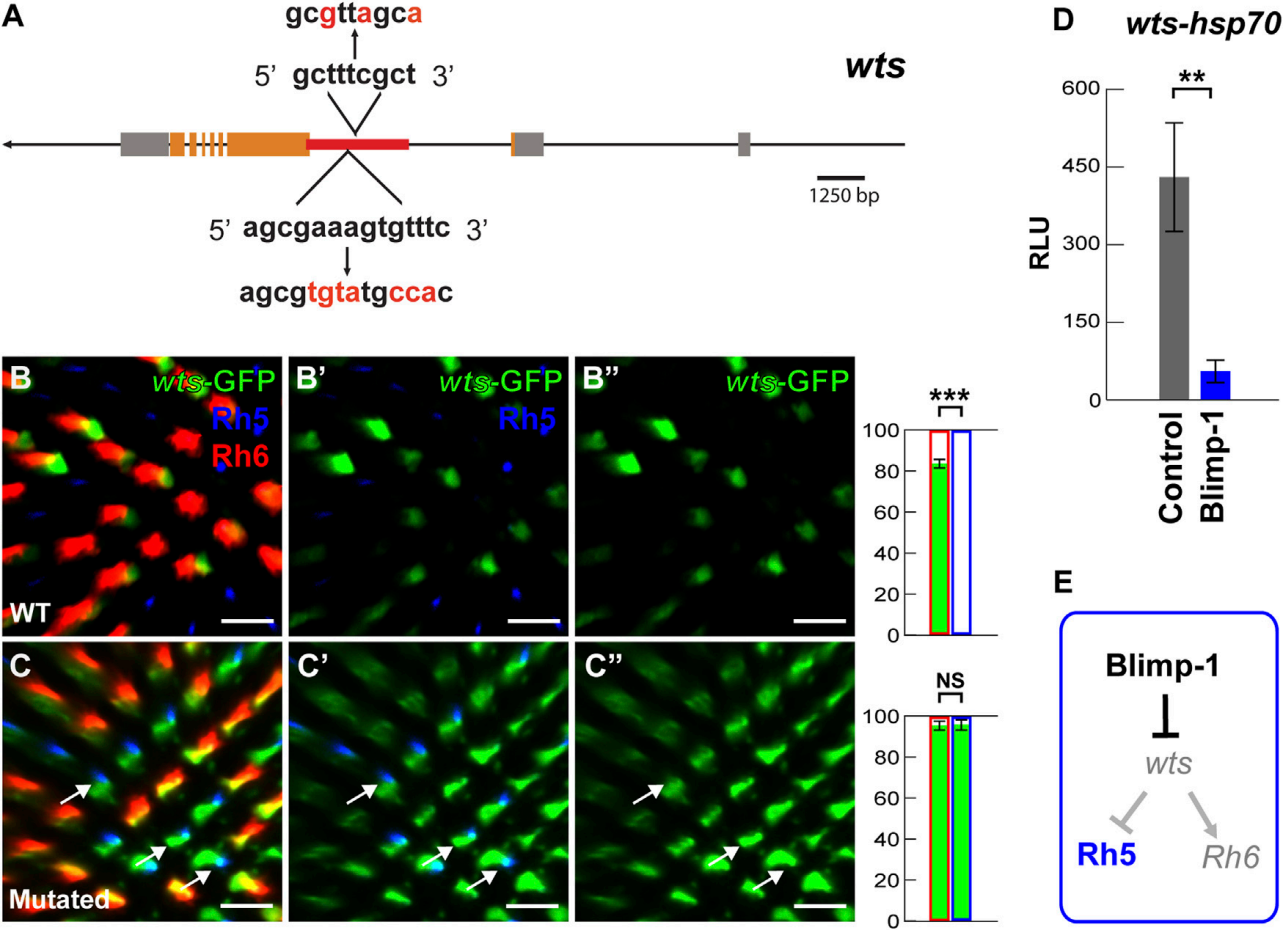
Blimp-1 represses the *warts* intronic enhancer. **(A)** Schematic of the *wts* locus. The red line indicates the location of the 2.6 kb *wts* enhancer in the second intron that contains two conserved Blimp-1 motifs. The red letters indicate introduced mutations. **(B–B’)** The 2.6 kb *wts* enhancer fused to an *hsp70* promoter drives GFP (hereafter referred to as *wts-*GFP reporter) in the majority of Rh6 photoreceptors. Right: percentage of Rh6 photoreceptors (red outline) or Rh5 photoreceptors (blue outline) that co-express GFP (green bars), respectively. %Rh6/GFP co-expression vs. %Rh5/GFP co-expression was compared with an ANOVA and a *post hoc* Tukey HSD Test; ***p < 0.0001. *N* = 5 retinas. **(C–C’)** Mutation of the two conserved Blimp-1 motifs causes *wts-*GFP reporter de-repression in Rh5 photoreceptors. White arrows indicate examples of *wts-*GFP de-repression. Right: percentage of Rh6 photoreceptors or Rh5 photoreceptors that co-express GFP, respectively. *N* = 5 retinas. All scale bars, 10 µm. **(D)** Dual luciferase reporter assay. The intronic *wts* reporter (*wts-hsp70-luc*) is active in S2 cells, but Blimp-1 strongly represses it (*N* = 3). *Y*-axis: Relative Luminescence Units (RLU) of *wts-hsp70-luc* normalized to the Renilla luciferase control reporter (see Materials and methods). **(E)** Schematic of Blimp-1 function in color photoreceptor specification: Blimp-1 represses *wts*/Rh6 fate to promote Rh5 fate.

Because the Hippo pathway is inactivated in the Rh5 PRs that require Blimp-1 for their specification (Mikeladze-Dvali et al., 2005; Jukam et al., 2013) and Blimp-1 is a transcriptional repressor (Keller and Maniatis, 1991; Yu et al., 2000; Agawa et al., 2007; Ozturk-Colak et al., 2018), we asked whether Blimp-1 represses the Hippo pathway in R8 PRs. A candidate target for Hippo pathway repression is its nexus *wts,* which is transcriptionally repressed in Rh5 PRs (Figure 2A) (Mikeladze-Dvali et al., 2005; Jukam et al., 2013; Pojer et al., 2021). We identified an enhancer in the second *wts* intron (Figure 3A; see Materials and methods) that was sufficient to recapitulate PR subtype-specific *wts* expression in the Rh6 PRs when fused to an *egfp* reporter gene (*wts-*GFP); this allowed us to test whether Blimp-1 is required to repress *wts* transcription in Rh5 PRs*.* Indeed, *wts-*GFP was de-repressed with Rh6 upon *Blimp-1* knockdown (Figures 2B–C′, I; Supplementary Figures S2A–C), suggesting that Blimp-1 is required for the transcriptional repression of *wts.* Since *melt* is expressed in Rh5 PRs and repressed by Wts in Rh6 PRs, we tested if Blimp-1 is also required for *melt* activation in Rh5 PRs. Indeed, *melt* transcriptional reporter expression (Figures 2D, D′) was lost when *Blimp-1* was knocked down (Figures 2E, E’, 2I). Taken together, these data suggest that Blimp-1 is required for both the repression of *wts* and the expression of *melt*.

The *wts* and *melt* reporter results suggest that Blimp-1 acts genetically upstream of *wts*. To test this hypothesis, we performed epistasis experiments. Indeed, RNAi-mediated knockdown of *wts* caused a gain of Rh5 and loss of Rh6 (Figure 2F) even with concomitant knockdown of *Blimp-1* (Figures 2G,J)*.* Since *wts* knockdown reverses the *Blimp-1* knockdown phenotype (gain of Rh6 and loss of Rh5), this provides further support that Blimp-1 acts upstream of *wts*. To corroborate this result, we analyzed *merlin (mer)* mutant clones. Mer is a FERM domain-containing protein that is required for Wts activity in R8 PRs, and *mer* mutant clones exclusively express Rh5 (Jukam and Desplan, 2011). Likewise, we found that *mer; Blimp-1* double mutants also exclusively expressed Rh5 (Figure 2H, H’). In summary, the perturbation of Wts activity, either through RNAi-mediated knockdown of *wts* or mutation of *mer*, reverses the *Blimp-1* mutant phenotype. These data show that Blimp-1 acts genetically upstream of w*ts.*

Lastly, we investigated the possibility that Blimp-1 directly represses *wts* transcription. We identified two conserved motifs (Supplementary Figure S3A) in the *wts* intronic enhancer that match the Blimp-1 consensus motif AGNGAAAG (Kuo and Calame, 2004; Ancelin et al., 2006; Katoh et al., 2010) as well as the *Drosophila* Blimp-1 Position Weight Matrix (Zhu et al., 2011) (Figure 3A) (see Materials and methods). Strikingly, the mutation of the conserved Blimp-1 motifs caused *wts-*GFP reporter de-repression in Rh5 PRs (compare Figures 3B-B”, C-C”), suggesting that the Blimp-1 motifs are required for *wts* repression in Rh5 PRs. Consistent with these *in vivo* data, Blimp-1 dramatically reduced (∼9-fold) *wts-hsp70-luc* reporter expression in *Drosophila* S2 cells (Figure 3D) (see Material and methods). Taken all the data together, we propose that Blimp-1 represses Rh6 fate through repression of *wts*, the nexus of the Hippo pathway, and thereby promotes Rh5 fate (Figure 3E).

### 3.4 The role of Blimp-1 in wing growth is independent of the hippo pathway

**FIGURE 4:**
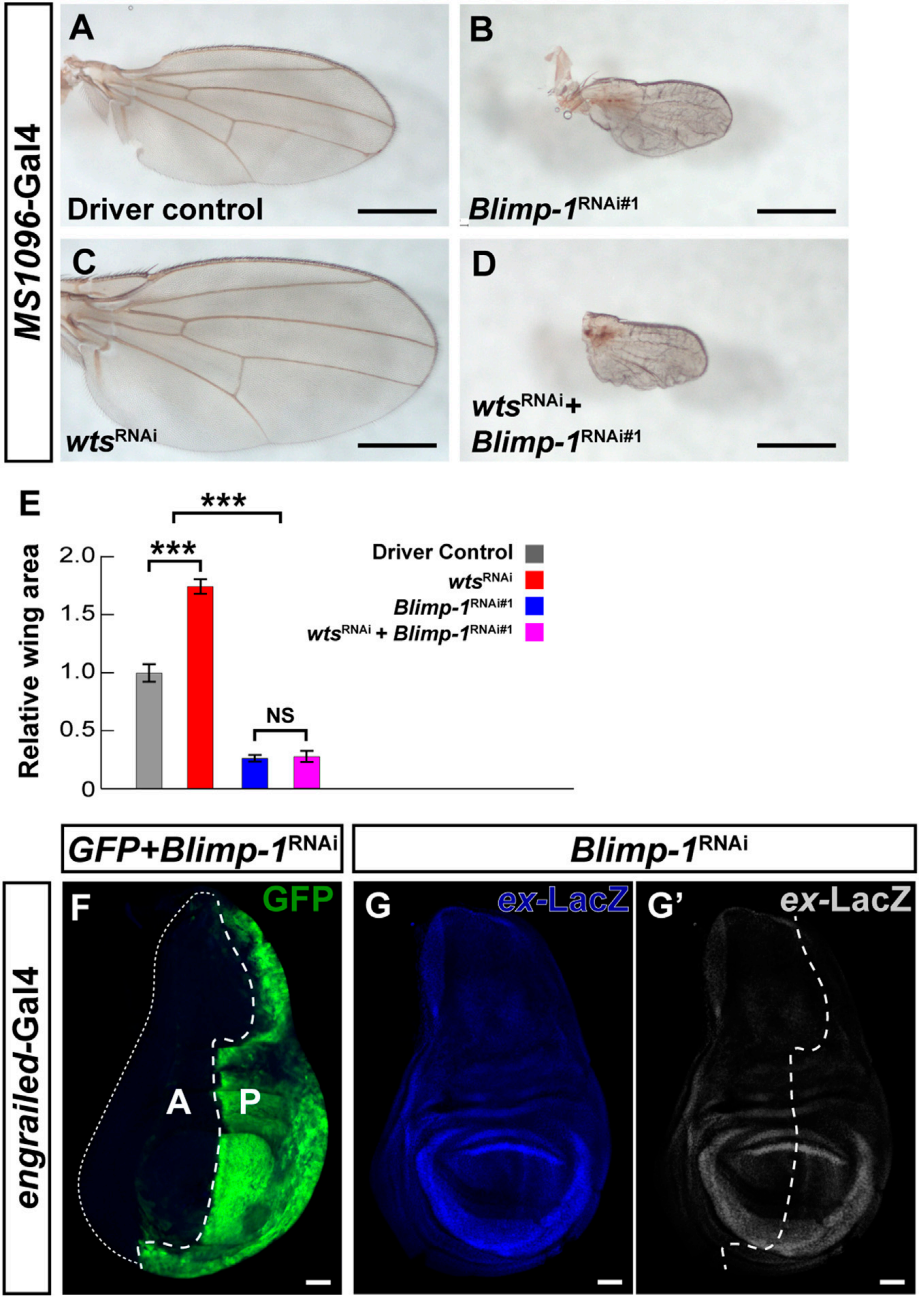
Blimp-1 plays a role in wing development independent of the Hippo pathway. **(A–D)** Wings of 2–4 days-old female flies raised at 25°C. Scale bars, 500 µm. **(A)** Wing disc driver control *MS1096*-*Gal4*/+. **(B)** *Blimp-1* knockdown causes a dramatic reduction in wing size. **(C)** *wts* knockdown causes an increase in wing size. **(D)** Knockdown of both *Blimp-1* and *wts* resembles the *Blimp-1* knockdown. **(E)** Quantification of wing area for each genotype normalized to the *MS1096*-*Gal4* driver control. Relative wing areas were compared with an ANOVA and a *post hoc* Tukey HSD Test; ***p < 0.0001. 6-8 wings were scored for each genotype. **(F)** Confocal image of a third instar larval wing disc with *engrailed-Gal4* driving GFP and *Blimp-1* ^*RNAi#1*^ in the posterior half. Blimp-1 knockdown does not appear to affect the size of the posterior half of the wing disc (marked by GFP) compared to the anterior half of the wing disc (GFP negative). White dashed line encircles the anterior half of the wing disc. Anterior is labeled with “A”, and posterior is labeled with “P”. **(G)** *Blimp-1* knockdown does not affect *expanded*-lacZ reporter expression in the posterior wing disc. **(G′)** *Expanded-*LacZ staining in grayscale. White dotted line indicates the approximate boundary between anterior and posterior halves based on the GFP staining in **(F)**. All scale bars for larval wing discs, 50 µm.

Since the canonical role of the Hippo pathway is to regulate organ growth (Halder and Camargo, 2013) and we found that Blimp-1 represses the core component *wts* in the post-mitotic PR context, we asked whether Blimp-1 regulates the Hippo pathway in mitotically active tissue. While *Blimp-1* knockdown caused a glossy eye phenotype (Wang et al., 2022) (Supplementary Figures S4A, B), it did not decrease the size of the adult eye (Supplementary Figures S4A, B), indicating that Blimp-1 does not regulate tissue growth in the developing eye. Moreover, knockdown of *wts* did not rescue the glossy eye phenotype (Supplementary Figure S4C **)**, indicating that this *Blimp-1* mutant phenotype is not related to the Hippo pathway. In addition, *Blimp-1* knockdown in the developing wing caused a dramatic decrease in adult wing size with two different wing disc drivers (Figures 4A, B; Supplementary Figures S4D, E). However, in contrast to the terminal PR differentiation context, and consistent with the glossy eye phenotype, the wing size defect could not be modified by concomitant knockdown of *wts* (Figures 4C–E). Moreover, knockdown of *Blimp-1* in the posterior half of the wing disc with *engrailed-Gal4* did not have any obvious effects on the size of the third instar larval wing disc in comparison to the anterior half (Figure 4F), suggesting that Blimp-1 likely regulates wing development during pupal stages, similar to its pupal role in post-mitotic PRs. Again, in contrast to post-mitotic PRs, this other role of Blimp-1 does not appear to involve regulation of Hippo pathway activity: *expanded-*LacZ (Yu and Pan, 2018), a transcriptional reporter of Yki activity, was unaffected when *Blimp-1* was knocked down in the posterior half of the developing wing disc (Figures 4G, G’). Furthermore, the intronic *wts* enhancer that is repressed by Blimp-1 in post-mitotic PRs was not detectable in the wing disc (Supplementary Figure S4F). Together, these data suggest that Blimp-1 is required for proper wing growth, but this function appears to be independent of the Hippo pathway.

### 3.5 Ecdysone signaling is cell autonomously required for blue-sensitive photoreceptor fate

**FIGURE 5:**
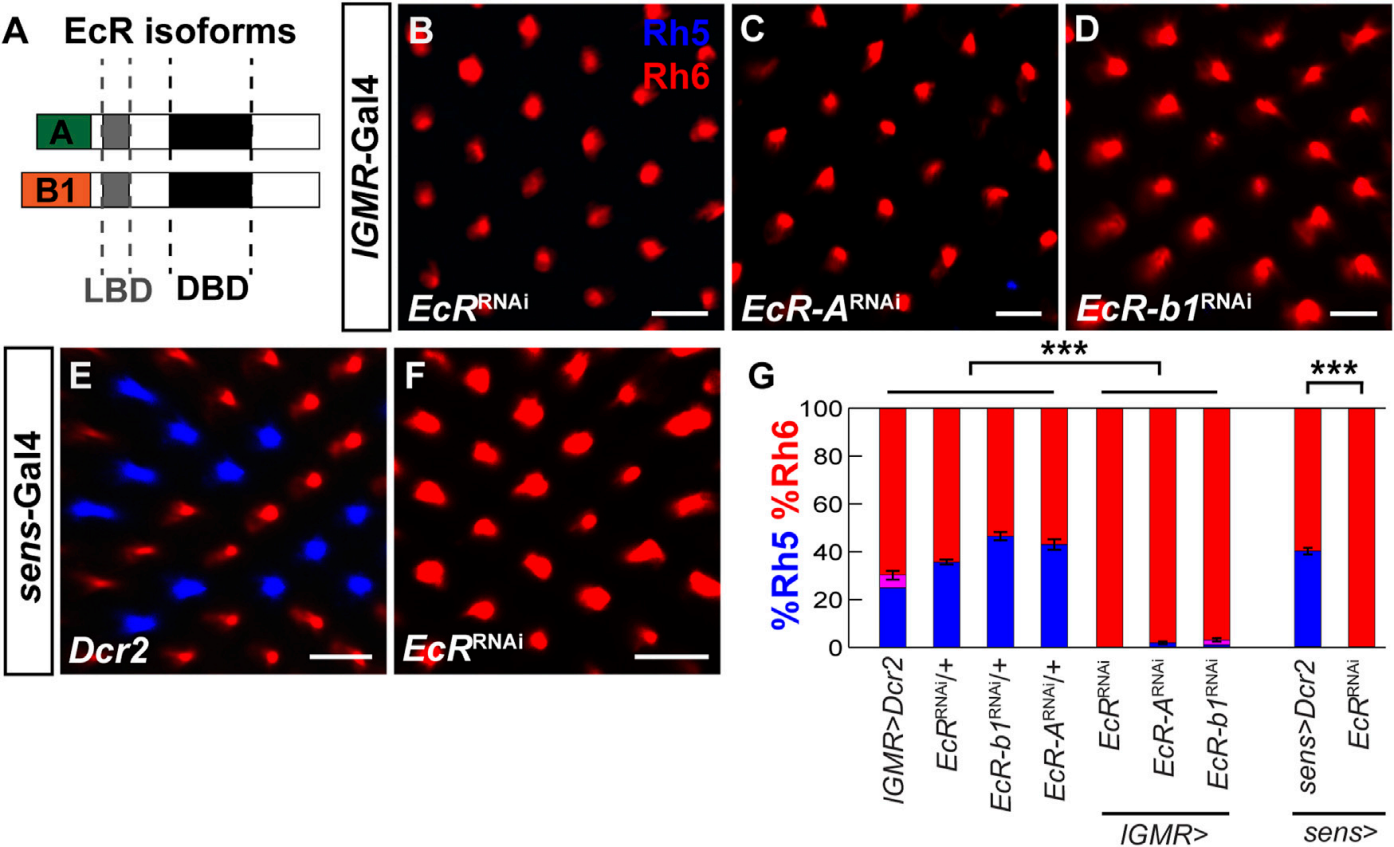
EcR is required cell autonomously to specify Rh5 fate. **(A)** Schematic of two EcR isoforms, EcR-A and EcR-b1, which contain identical DNA binding domains (DBD) and ligand binding domains (LBD) but differ in their N-terminal A/B domains. **(B)** Pan-photoreceptor knockdown of all *EcR* isoforms causes a loss of Rh5 and a gain of Rh6. **(C, D)** Pan-photoreceptor knockdown of the individual *EcR* isoforms *EcR-A* **(C)** and *EcR-b1* **(D)** causes a loss of Rh5 and gain of Rh6. **(E)** Expression of *Dcr2* with the R8 driver *sens-Gal4* at 29°C does not affect the Rh5:Rh6 ratio. **(F)** Expression of *EcR* ^RNAi^ and *Dcr2* with *sens-Gal4* at 29°C causes a loss of Rh5 and gain of Rh6. All scale bars, 10 µm. **(G)** Quantification of R8 subtypes in controls vs. *EcR* knockdowns. Mean %Rh6 was compared among genotypes with an ANOVA and a *post hoc* Tukey HSD Test; ***p < 0.0001. 5-8 retinas were scored for each genotype.

Blimp-1 is activated by the steroid hormone ecdysone (Agawa et al., 2007; Akagi and Ueda, 2011; Akagi et al., 2016; Ozturk-Colak et al., 2018) and Blimp-1 expression in the pupal retina requires the ecdysone receptor (EcR) (Wang et al., 2022). Therefore, we analyzed whether ecdysone signaling is required to specify Rh5 fate by performing RNAi-mediated knockdown of *EcR* with *lGMR-Gal4*. Closely resembling the *Blimp-1* mutant phenotype, *EcR* knockdown with an RNAi construct that targets all *EcR* isoforms caused a complete loss of Rh5 PRs and a gain of Rh6 PRs (Figures 5A,B). Since there are three EcR isoforms (EcR-A, EcR-b1, and EcR-b2) that have identical DNA binding domains but differ in their N-terminal A/B domains that allow them to elicit differential transcriptional responses (Mouillet et al., 2001; Schubiger et al., 2003), we additionally performed isoform-specific knockdowns. The knockdown of *EcR-A* and *EcR-b1* (an RNAi line specifically targeting *EcR-b2* was not available) each caused a loss of Rh5 PRs and gain of Rh6 PRs (Figures 5C, D), respectively, suggesting that both isoforms are non-redundantly required to specify Rh5 fate. Similarly, *EcR* knockdown using the R8 PR driver s*ens-Gal4* also caused a loss of Rh5 PRs and gain of Rh6 PRs (Figures 5E, F). Taken together (Figure 5G), ecdysone signaling is cell autonomously required in R8 PRs to specify Rh5 fate and to repress Rh6 fate*.*

### 3.6 Hr3 acts cell autonomously in R8 photoreceptors to promote blue-sensitive photoreceptor fate

**FIGURE 6:**
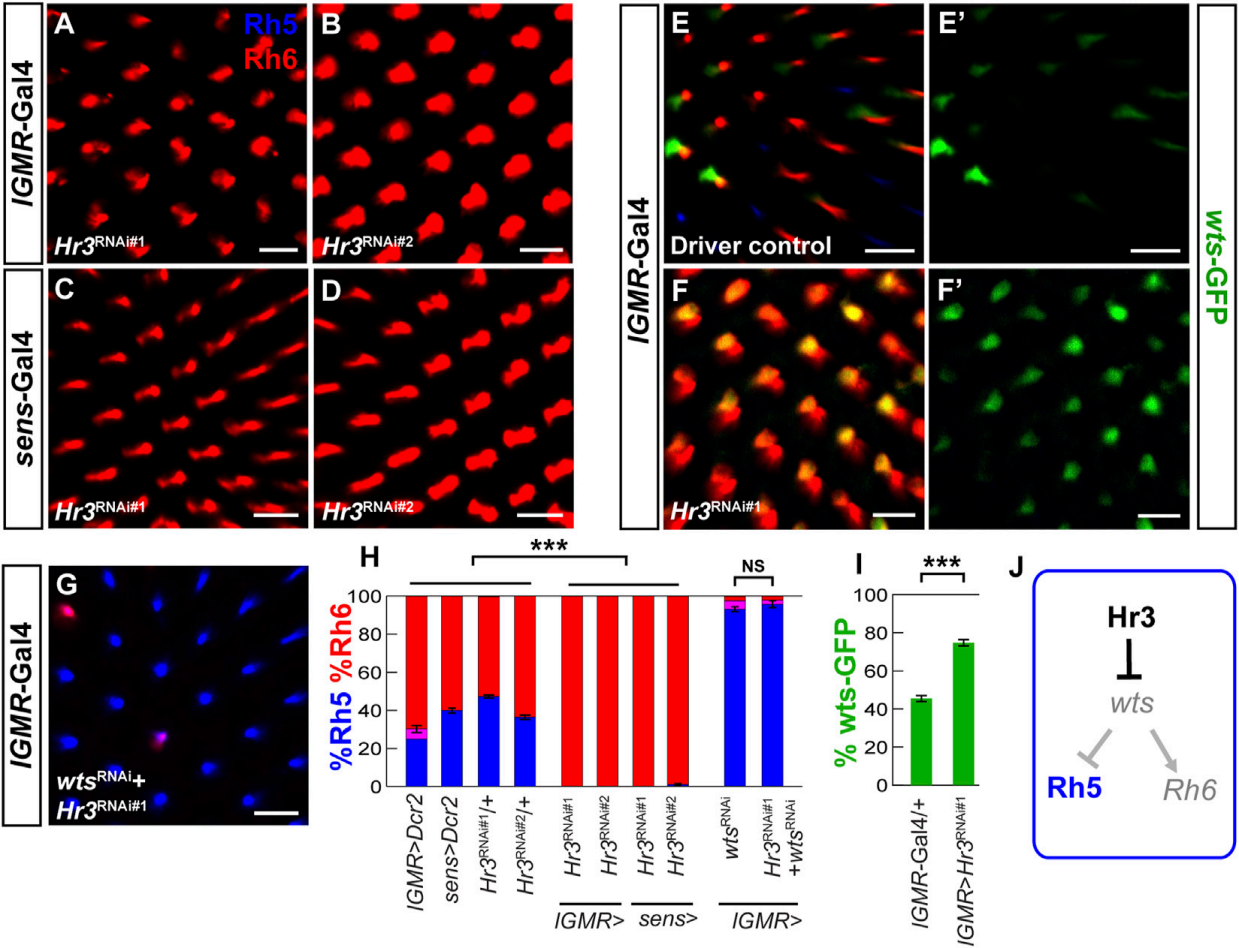
Hr3/RORβ is required to repress *warts* and to specify Rh5 fate.**(A,B)** Pan-photoreceptor knockdown of *Hr3* using two separate RNAi constructs, TRiP^#27253^ (*Hr3* ^*RNAi#1*^) and TRiP^#27254^ (*Hr3* ^*RNAi#2*^) causes a complete loss of Rh5 and gain of Rh6. **(C,D)** R8 photoreceptor knockdown with two separate RNAi constructs also causes a dramatic loss of Rh5 and gain of Rh6. **(E,E′)** In the heterozygous *lGMR-Gal4*/+ driver control, the *wts-*GFP transcriptional reporter is expressed in most Rh6 photoreceptors. **(F, F′)** Pan-photoreceptor knockdown of *Hr3* causes a de-repression of the *wts-*GFP reporter together with Rh6. **(G)** Pan-photoreceptor knockdown of both *Hr3* and *wts* causes a gain of Rh5 and loss of Rh6, resembling the *wts* knockdown phenotype. All scale bars, 10 µm. **(H)** Quantification of R8 subtypes in controls and *Hr3* knockdowns. Mean %Rh6 was compared among genotypes with an ANOVA and a *post hoc* Tukey HSD Test; ***p < 0.0001. 5-8 retinas were scored for each genotype. **(I)** Quantification of the GFP-expressing R8 photoreceptors in the driver control and *Hr3* knockdown. Mean %*wts-*GFP reporter expression in driver control vs. knockdown was compared with an ANOVA and a *post hoc* Tukey HSD Test; ***p < 0.0001. 6 retinas were analyzed for each genotype. **(J)** Schematic of Hr3 function in Rh5 photoreceptors: Hr3 represses *wts* and thereby promotes Rh5 fate.

In the developing mouse retina, the nuclear receptor RORβ and the transcription factor Otx2 activate Blimp-1 in retinal progenitor cells to repress bipolar interneuron fate and promote rod PR fate (Jia et al., 2009; Brzezinski et al., 2010; Katoh et al., 2010; Brzezinski et al., 2013; Wang et al., 2014; Goodson et al., 2020). Because the *Drosophila* ortholog of Otx2, Otd, is required for Rh5 fate (McDonald et al., 2010; Jukam et al., 2013), we asked whether the *Drosophila* ortholog of RORβ, the ecdysone-responsive Hormone Receptor 3 (Hr3) (Kageyama et al., 1997; Lam et al., 1999) is also required for Rh5 fate. Indeed, RNAi-mediated knockdown of *Hr3* with two different RNAi lines combined with the *lGMR-Gal4* driver (Figures 6A, B) or the *sens-Gal4* driver (Figures 6C, D) caused a nearly complete loss of Rh5 PRs and gain of Rh6 PRs. Next, we hypothesized that Hr3 represses the Hippo pathway in Rh5 PRs. Consistent with this hypothesis, *Hr3* knockdown caused a significant de-repression of the *wts-*GFP reporter (Figures 6E–F’) and concomitant knockdown of *wts* reversed the *Hr3* knockdown phenotype **(** Figure 6G **)**. As expected from the gain of *wts*, *Hr3* knockdown also caused a loss of *melt-*GFP reporter expression (Supplementary Figures S5A–C). Moreover, Hr3 significantly reduced *wts-hsp70-luc* reporter expression in *Drosophila* S2 cells, albeit to a lesser extent (∼3 fold) than Blimp-1 (Supplementary Figure SD). In summary (Figures 6H, I), these data suggest that both Blimp-1 and Hr3 are cell autonomously required to specify Rh5 fate in R8 PRs by repressing *wts* (Figure 6J).

**FIGURE 7:**
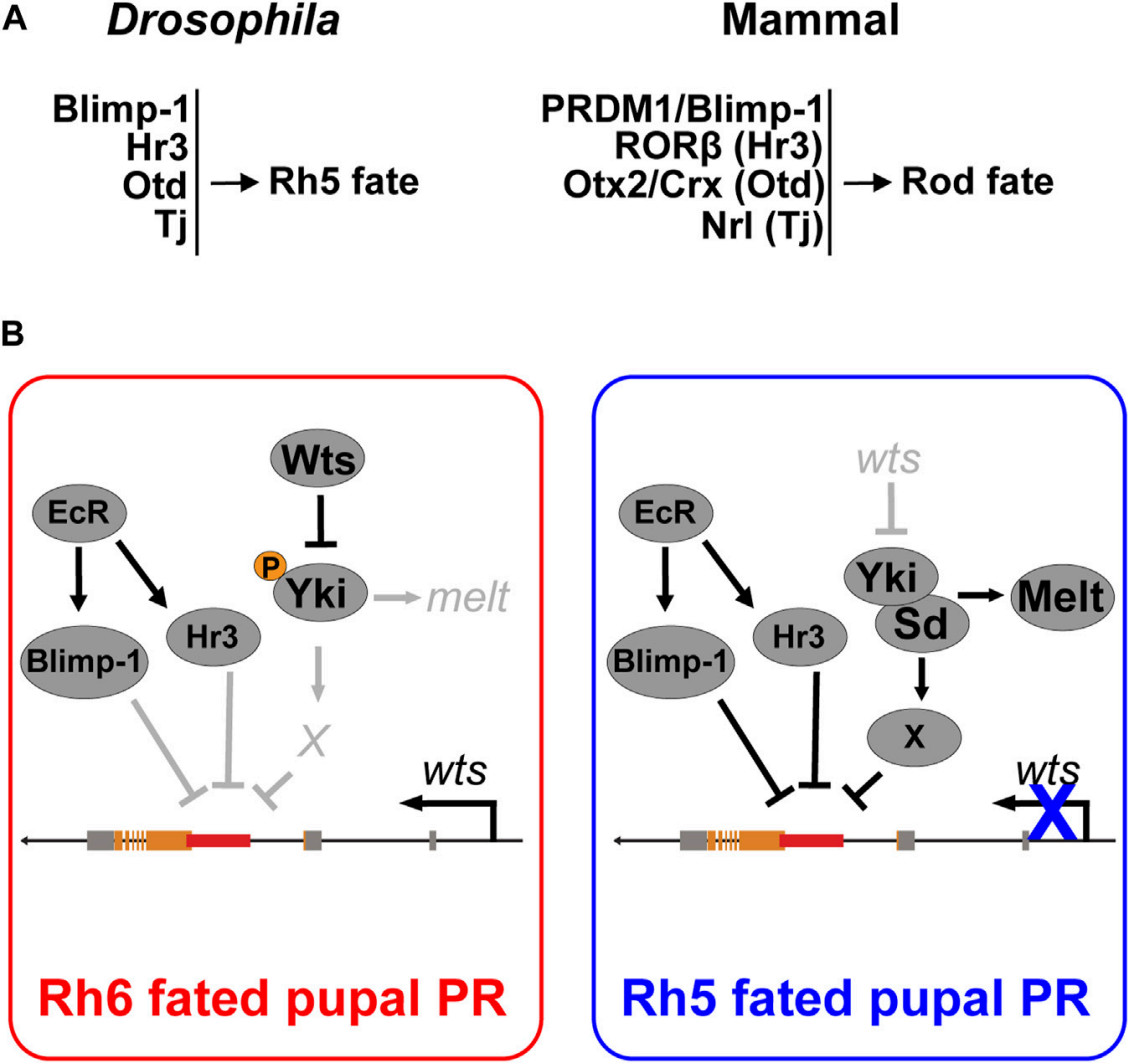
Cell fate decisions in the *Drosophila* and mammalian retina. **(A)** Summary of conserved transcription factors that are necessary to specify Rh5 photoreceptor fate in *Drosophila* and rod photoreceptor fate in mammals. **(B)** Model for Blimp-1’s function as a permissive factor that represses *wts* at the transcriptional level in parallel with the ecdysone-responsive Hr3. Blimp-1 is expressed in pupal PRs in an EcR-dependent manner, but when Yki is not activated, Blimp-1 is not sufficient to repress *wts* and thereby gives rise to Rh6 fate. However, when Yki is transiently activated, possibly in response to the TGFβ signal from the distal R7 photoreceptor, Yki/Sd activate an unknown Rh5 subtype-specific transcription factor “X” that acts in combination with Blimp-1 and Hr3 to repress the *wts* enhancer, thereby silencing *wts* transcription and giving rise to robust Yki activation as well as Rh5 fate.

We next asked whether Hr3 is required for Blimp-1 expression. To this end, we performed RNAi-mediated knockdown of *Hr3* and assessed the expression of Blimp-1 in pupal PRs. While *Blimp-1* knockdown abolished Blimp-1 expression, *Hr3* knockdown did not affect Blimp-1 expression compared to the driver control (Supplementary Figures S5E–G’). Therefore, Hr3 is not required for Blimp-1 expression in pupal PRs. Taken together, these data suggest that Hr3 promotes Rh5 fate by acting in parallel with Blimp-1 to repress *wts* (Figure 7B).

## 4 Discussion

### 4.1 Conserved transcription factors control binary cell fate decisions in the mammalian and the *Drosophila* retina

Here, we analyzed the regulatory mechanisms that specify two related *Drosophila* PR subtypes that express different color-sensing pigments (Rh5 or Rh6) in a mutually exclusive manner. We discovered that the *Drosophila* orthologs of the mammalian rod PR fate determinants Blimp-1/PRDM1 (Brzezinski et al., 2010; Katoh et al., 2010; Brzezinski et al., 2013; Goodson et al., 2020) and Hr3/RORβ (Wang et al., 2014) also play a role in terminal *Drosophila* PR specification, but in the cone-equivalent “inner” R8 PRs rather than the rod-equivalent “outer” R1-R6 PRs. In the binary R8 PR subtype decision, Blimp-1 and Hr3 promote the blue-sensitive/Rh5 PR fate and repress the green-sensitive/Rh6 PR fate by repressing *wts* and activating *melt*.

A previous study had unraveled that Otd, the ortholog of the mammalian PR fate determinants Otx2 and Crx (McDonald et al., 2010), and Tj, the ortholog of the mammalian rod fate determinant Nrl, are also required to specify Rh5 fate (Jukam et al., 2013). Otd and Tj form a coherent feedforward loop that allows Yki/Sd to activate *melt* and to repress *wts* (Jukam et al., 2013)*.* In the mammalian retina, the Otd and Tj orthologs Otx2/Crx and Nrl, respectively, promote rod PR fate: Otx2 and Crx are both necessary for the expression of Nrl (Montana et al., 2011; Roger et al., 2014), which is necessary and sufficient for rod PR fate (Mears et al., 2001; Oh et al., 2007). *NRL* mutations have been associated with retinitis pigmentosa (Bessant et al., 1999) and mutations in Blimp-1, RORβ, Otx2, Crx, or Nrl are associated with a loss of rod PRs in mammals (Mears et al., 2001; Nishida et al., 2003; Daniele et al., 2005; Koike et al., 2007; Jia et al., 2009; Katoh et al., 2010). Likewise, loss of Blimp-1, Hr3, Otd, or Tj are each associated with a loss of blue-sensing PR fate (this study) (Jukam et al., 2013). Although the *Drosophila* eye and the mammalian eye seem to use different mechanisms for eye and PR development (Rister and Desplan, 2011; Cepko, 2015; Eldred et al., 2018), our current study and previous results (Jukam et al., 2013) suggest that a conserved set of transcription factors is used in both animal groups for specific binary PR fate decisions (Figure 7A).

While Blimp-1/PRDM1 and Hr3/RORβ promote rod fate in mammals and blue-sensitive/Rh5 PR fate in *Drosophila* respectively, the mechanisms by which they regulate these cell fate decisions differ: in mammals, Hr3/RORβ is required to activate *Blimp-1/PRDM1* to repress bipolar fate and specify rod fate (Wang et al., 2014). However, in developing *Drosophila* PRs, Hr3 does not regulate *Blimp-1* expression (Supplementary Figures SE–G’) but rather acts in parallel with Blimp-1 to repress *wts*. Given that conserved Blimp-1 motifs are required to repress *wts* in blue-sensitive/Rh5 PRs and Blimp-1 represses a *wts-hsp70-luc* reporter *in vitro* (Figures 3B–D), it is likely that Blimp-1 represses the *wts* enhancer directly. Since we did not find conserved Hr3 motifs in the *wts* enhancer, future studies will have to analyze the *in vivo* relevance of the Hr3-mediated *wts* repression that we found in cultured cells.

### 4.2 Blimp-1 acts as a permissive factor to promote blue-sensitive photoreceptor fate

Blimp-1/PRDM1 controls cell fate decisions in diverse developmental contexts (Bikoff et al., 2009). In pupal *Drosophila* PRs, Blimp-1 expression is ecdysone-dependent and regulates the terminal differentiation of the eye non-autonomously in non-neuronal cells (Wang et al., 2022). In the current study, we revealed a novel cell autonomous role of Blimp-1 in the terminal differentiation of color-sensing PR neurons. Blue-sensitive PR fate requires ecdysone signaling (EcR) and the ecdysone-responsive regulators Blimp-1 and Hr3, which both act to promote Rh5 PR fate. In contrast, in the larval fat body, Blimp-1 and Hr3 play antagonistic roles in regulating the regulatory gene *ftz-f1* to control pupation timing: Blimp1 represses *ftz-f1*, while Hr3 activates *ftz-f1* (Kageyama et al., 1997; Lam et al., 1999; Agawa et al., 2007).

The R8 PR specification network involves several permissive transcription factors that are not restricted to one of the two subtypes. Blimp-1 is transiently expressed during the early differentiation of both R8 PR subtypes, where it on the one hand represses *wts* in Rh5-fated PRs but on the other hand permits *wts* expression in Rh6-fated PRs. Likewise, Otd and Tj are expressed in both Rh5- and Rh6-fated PRs and act as permissive factors for Rh5 fate. Yki/Sd are unable to activate *melt* in the absence of Otd or Tj, or to activate *Rh5* in the absence of Otd (Jukam et al., 2013). However, Tj and Otd are not sufficient to activate *melt* in the absence of Yki or Sd, and Otd is not sufficient to activate *Rh5* (Jukam et al., 2013)*.* Therefore, the conserved PR fate specification module is required to establish a post-mitotic context wherein *melt* and *wts* can function as a bi-stable switch to rewire the Hippo pathway. The proposed context-specificity is consistent with the finding that Blimp-1 represses *wts* in the post-mitotic PR context, but not in the wing growth context, and that the intronic *wts* enhancer drives expression in Rh6 PRs but not in the wing disc.

Since Blimp-1 acts as a permissive factor that represses *wts* in Rh5-fated PRs, a possible regulatory scenario is that an unknown transcription factor is specifically expressed in the Rh5-fated PRs and acts in combination with Blimp-1 to repress *wts*, analogous to how Yki/Sd activate *melt* in combination with Otd and Tj. Since Yki is active in Rh5 PRs but not in Rh6 PRs (Jukam et al., 2013), another possibility is that Yki is transiently activated in Rh5-fated pupal PRs, and then acts in combination with Blimp-1 to repress *wts* transcriptionally, permanently inactivating the Hippo pathway to give rise to robust Yki activation and Rh5 fate. Alternatively, we propose a scenario that contains an intermediate step (Figure 7B) in which Yki/Sd activate an additional transcription factor that acts with Blimp-1 to repress *wts* in a combinatorial manner.

In conclusion, the *Drosophila* color PR subtype specification is an excellent model to study how terminal cell fate decisions mediate the differential expression of sensory receptor proteins in related subsets of sensory neurons. The analysis of the underlying mechanisms gives insights into how conserved transcription factors generate sensory neuron diversity and potentially inform treatments for diseases that affect specific sensory neuron types.

## Data Availability

The original contributions presented in the study are included in the article/Supplementary Material, further inquiries can be directed to the corresponding author.

## Supplementary material

SUPPLEMENTARY FIGURE S1 Blimp-1 acts in mid-pupal R8 photoreceptors. **(A-F’)** Confocal images of whole mounted retinas from different pupal timepoints stained with antibodies for Elav (red, photoreceptor nuclei) and Blimp-1 (blue). **(A,A’)** Blimp-1 is weakly expressed in photoreceptors at 24 h after puparium formation. Note the partial overlap with the Elav nuclear marker (white arrow). Inset shows ommatidium of the arrowed photoreceptor nucleus. **(B,B’)** Blimp-1 is strongly expressed at 48 h after puparium formation. White arrow indicates partial Blimp-1/Elav overlap; inset shows ommatidium of the arrowed photoreceptor nucleus. **(C,C’)** Blimp-1 is absent in R8 photoreceptors at 72 h after puparium formation. **(D,D’)** Blimp-1 is absent in adult R8 photoreceptors. **(E,E’)** Blimp-1 expression at 48 h after puparium formation in the heterozygous *lGMR-Gal4*; *UAS-Dcr2* driver control. Note individual strong Blimp-1-positive nuclei (one example is indicated by white arrow). **(F,F’)** Pan-photoreceptor knockdown of *Blimp-1* causes a loss of Blimp-1 staining. **(G–I,K,L)** Confocal images of whole mounted retinas from adult males raised at 29^°^C. **(G)** In driver control males with hemizygous *UAS-Dcr2* on the X chromosome and heterozygous *sens-Gal4* on the third chromosome, the ratio of Rh5:Rh6 is normal. **(H)** *Blimp-1* knockdown with two copies of *UAS*-*Blimp-1* ^*RNAi#1*^, one copy of *sens-Gal4*, and hemizygous *UAS-Dcr2* causes a loss of Rh5 and gain of Rh6. **(I)** The Rh5:Rh6 ratio is normal in the homozygous *UAS*-*Blimp-1* ^*RNAi#1*^ control. **(J)** Quantification of R8 subtypes in controls and *Blimp-1* knockdown. Graph shows %R8 photoreceptors that express Rh5 exclusively (blue), Rh6 exclusively (red), or co-express Rh5 and Rh6 (magenta). Mean %Rh6 was compared among genotypes with an ANOVA and a post-hoc Tukey HSD Test; *** p<0.0001. 8 retinas were scored for each genotype. **(K)** Pan-photoreceptor expression of a constitutively active form of Babo (Babo*) causes a gain of Rh5 and loss of Rh6. **(L)** Pan-photoreceptor *Blimp-1* knockdown causes a loss of Rh5 and gain of Rh6 even with concomitant ectopic Babo* expression. All scale bars, 10 µm.

SUPPLEMENTARY FIGURE S2 Blimp-1 knockdown de-represses *wts*-GFP. **(A)** Longitudinal section of the heterozygous *lGMR-Gal4* driver control with a *wts-*GFP transcriptional reporter. Note that *wts-*GFP overlaps with Rh6 and not with Rh5; white arrow indicates an Rh6-expressing photoreceptor that does not express *wts-*GFP, suggesting that the heterozygous *wts* reporter is not strong enough to label all Rh6 photoreceptors. **(A’)** Rh5 channel. **(A’’)** Rh6 channel. **(A’’’)** *wts-*GFP channel (grayscale). **(B)** Longitudinal section of *Blimp-1* ^*RNAi#1*^ expressed with *lGMR-Gal4* in the presence of a *wts-*GFP transcriptional reporter. There is a loss of Rh5 and gain of Rh6, and *wts-*GFP expands with Rh6. Morphological defects caused by *Blimp-1* knockdown made it difficult to observe the *wts-*GFP reporter; longitudinal sections circumvented this issue. Similar to the driver control, there were occasional instances of Rh6-expressing photoreceptors that did not express *wts-*GFP (white arrow). **(B’)** Rh5 channel. **(B’’)** Rh6 channel. **(B’’’)** *wts-*GFP channel (grayscale). **(C)** Quantification of the percentage of Rh6 photoreceptors (red outline) that co-express GFP (green bars) in the *lGMR-Gal4* driver control and *Blimp-1* knockdown, respectively. %*wts-*GFP/Rh6 co-expression was compared for the two genotypes with an ANOVA and a post-hoc Tukey HSD Test; *** p<0.0001. *N* = 6 retinas.

SUPPLEMENTARY FIGURE S3 Conservation analysis of Blimp-1 motifs in the *wts* intron. **(A)** Alignments of the two Blimp-1 motif regions located in the intronic *wts* enhancer of ten *Drosophila* species. Blue indicates parts of the Blimp-1 motifs matching the consensus AGNGAAAG.

SUPPLEMENTARY FIGURE S4 Blimp-1 does not regulate *wts* in a growth context. **(A,B)** Knockdown of *Blimp-1* using the eye driver *GMR-Gal4* did not affect adult eye size compared to the heterozygous driver control. Note the glossy eye phenotype caused by *Blimp-1* knockdown due to its role in corneal lens formation (Wang et al., 2022). **(C)** Concomitant knockdown of *wts* with *Blimp-1* using *GMR-Gal4* did not affect the glossy eye phenotype that is caused by *Blimp-1* knockdown. **(D,E)** Knockdown of *Blimp-1* using the wing disc driver *nubbin-Gal4* causes a dramatic decrease in wing size compared to the heterozygous driver control. Scale bars, 500 µm **(E)** The intronic *wts-*GFP reporter that is expressed in post-mitotic photoreceptors is not active in the third instar larval wing disc. White dashed line indicates the edge of the wing disc. Scale bar, 50 µm.

Supplementary Figure S5 Hr3 regulates *melt* and *wts* but not Blimp-1. **(A)** In the heterozygous *lGMR-Gal4* driver control, *melt-*GFP is expressed in most Rh5 PRs and absent from Rh6 PRs. **(A’)** GFP channel. White arrows indicate examples of Rh5/*melt-*GFP co-expression. **(B)** Knockdown of *Hr3* using *lGMR-Gal4* causes a loss of *melt-*GFP. **(B’)** GFP channel. All scale bars, 10 µm. **(C)** Quantification of the GFP-expressing R8 photoreceptors in the driver control and *Hr3* knockdown. Mean %*melt-*GFP reporter expression in driver control *vs.* knockdown was compared with an ANOVA and a post-hoc Tukey HSD Test; *** p<0.0001. 6 retinas were analyzed for each genotype. **(D)** Dual luciferase reporter assay. The intronic *wts* reporter (*wts-hsp70-luc*) is active in S2 cells, but Hr3 represses it (*N* = 3). *Y*-axis: Relative Luminescence Units (RLU) of *wts-hsp70-luc* normalized to the Renilla luciferase control reporter (see Materials and methods). **(E-G’)** Pupal retinas with *lGMR-Gal4* at 48 h after puparium formation stained for Blimp-1 (blue) and the nuclear marker Elav (red). **(E)** The heterozygous *lGMR-Gal4*; *UAS-Dcr2* driver control shows Blimp-1 expression with occasional strong nuclear localization. White arrow indicates nuclear Blimp-1. **(E’)** Blimp-1 channel. **(F)** Pan-photoreceptor knockdown of *Blimp-1* abolishes Blimp-1 signal. **(F’)** Blimp-1 channel. **(G)** Pan-photoreceptor knockdown of *Hr3* does not affect Blimp-1 expression. Moreover, there was occasional strong nuclear localization, similar to the driver control, suggesting that Hr3 is neither required for Blimp-1 expression, nor its nuclear localization. White arrow indicates nuclear Blimp-1. **(G’)** Blimp-1 channel.
