# Supplementary figures and images for: Blimp-1/PRDM1 and Hr3/RORβ specify the blue-sensitive photoreceptor subtype in Drosophila by repressing the hippo pathway
Source: Front Cell Dev Biol. 2023 Mar 7;11:1058961. doi: 10.3389/fcell.2023.1058961 (PMC10027706; doi:10.3389/fcell.2023.1058961)

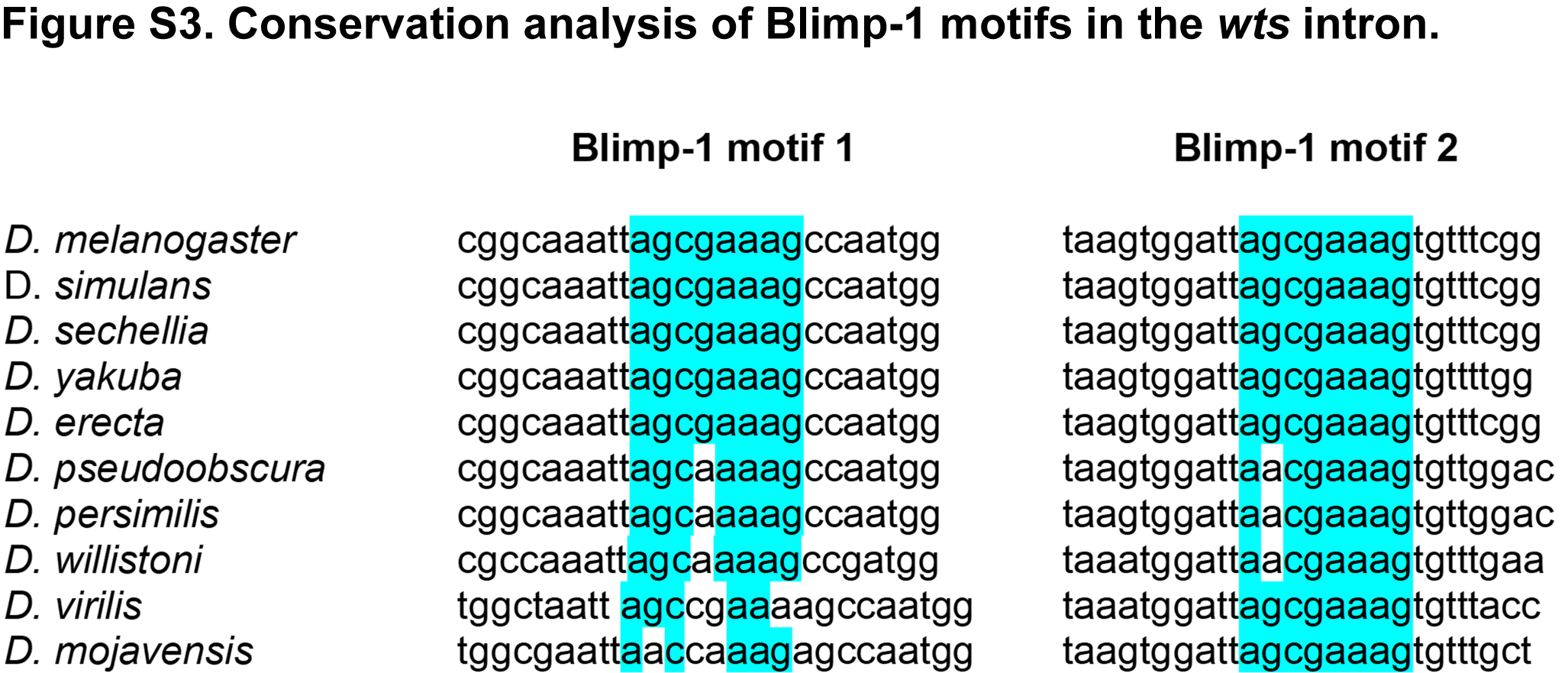

Supplement: Supplementary file 1 [file Image3.TIF]

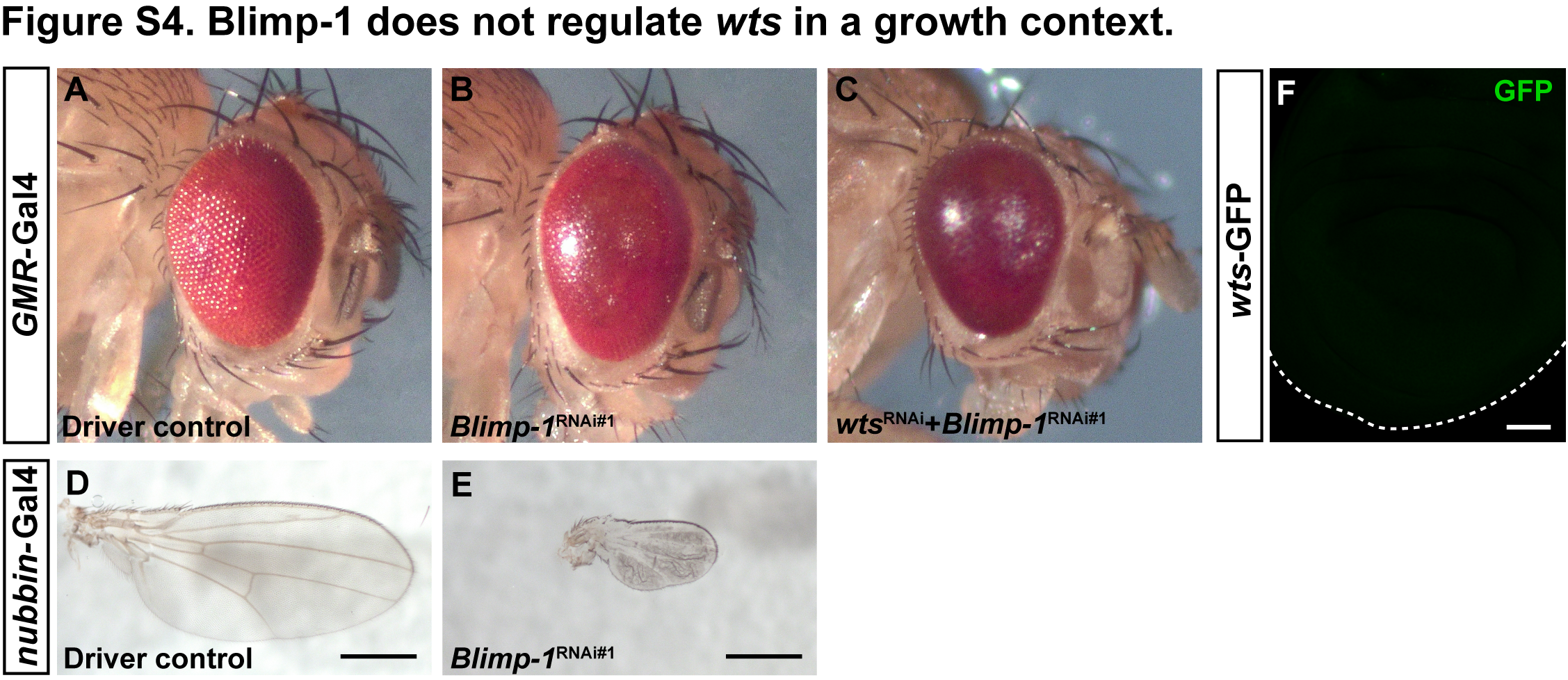

Supplement: Supplementary file 2 [file Image4.TIF]

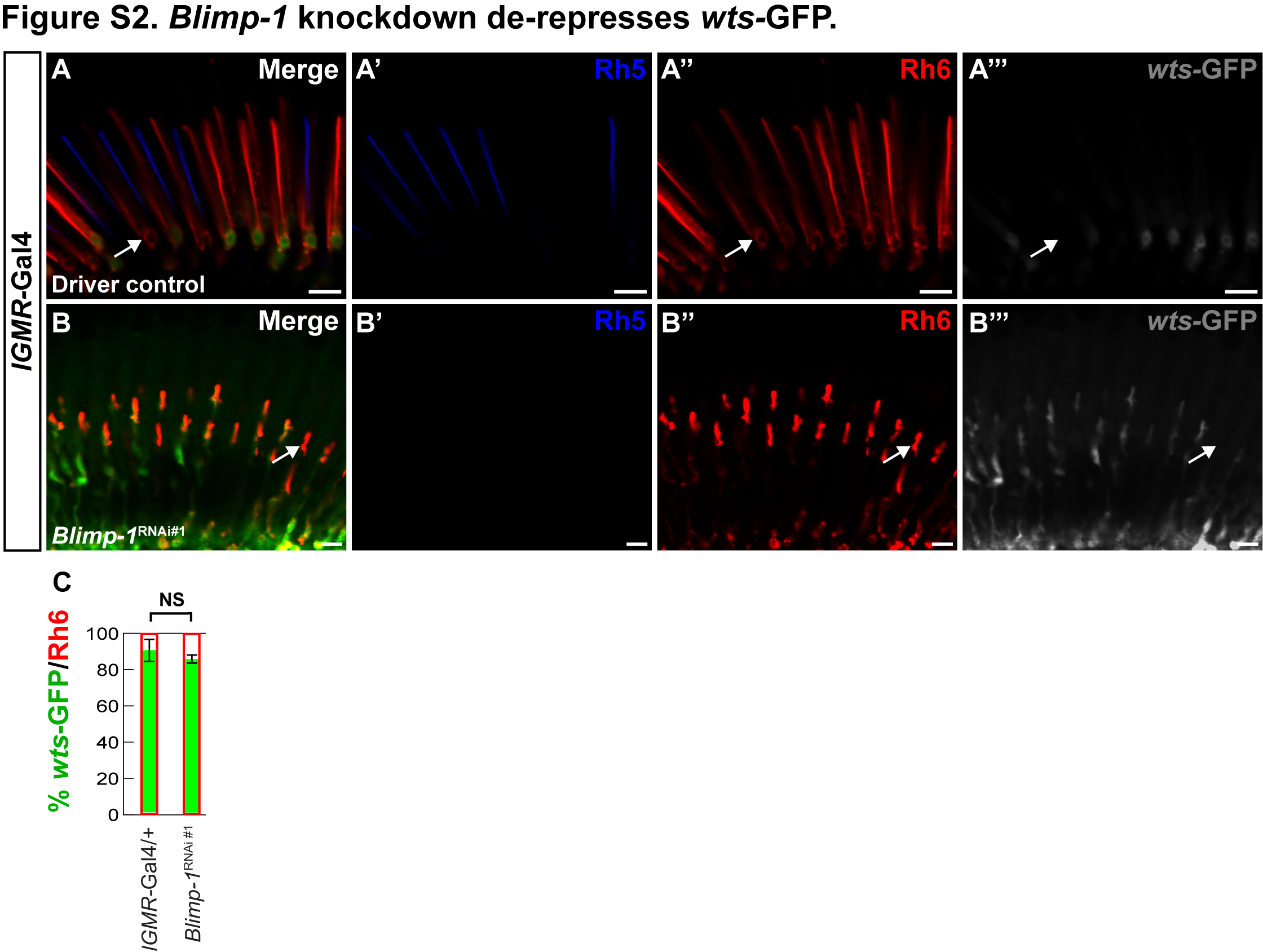

Supplement: Supplementary file 3 [file Image2.TIF]

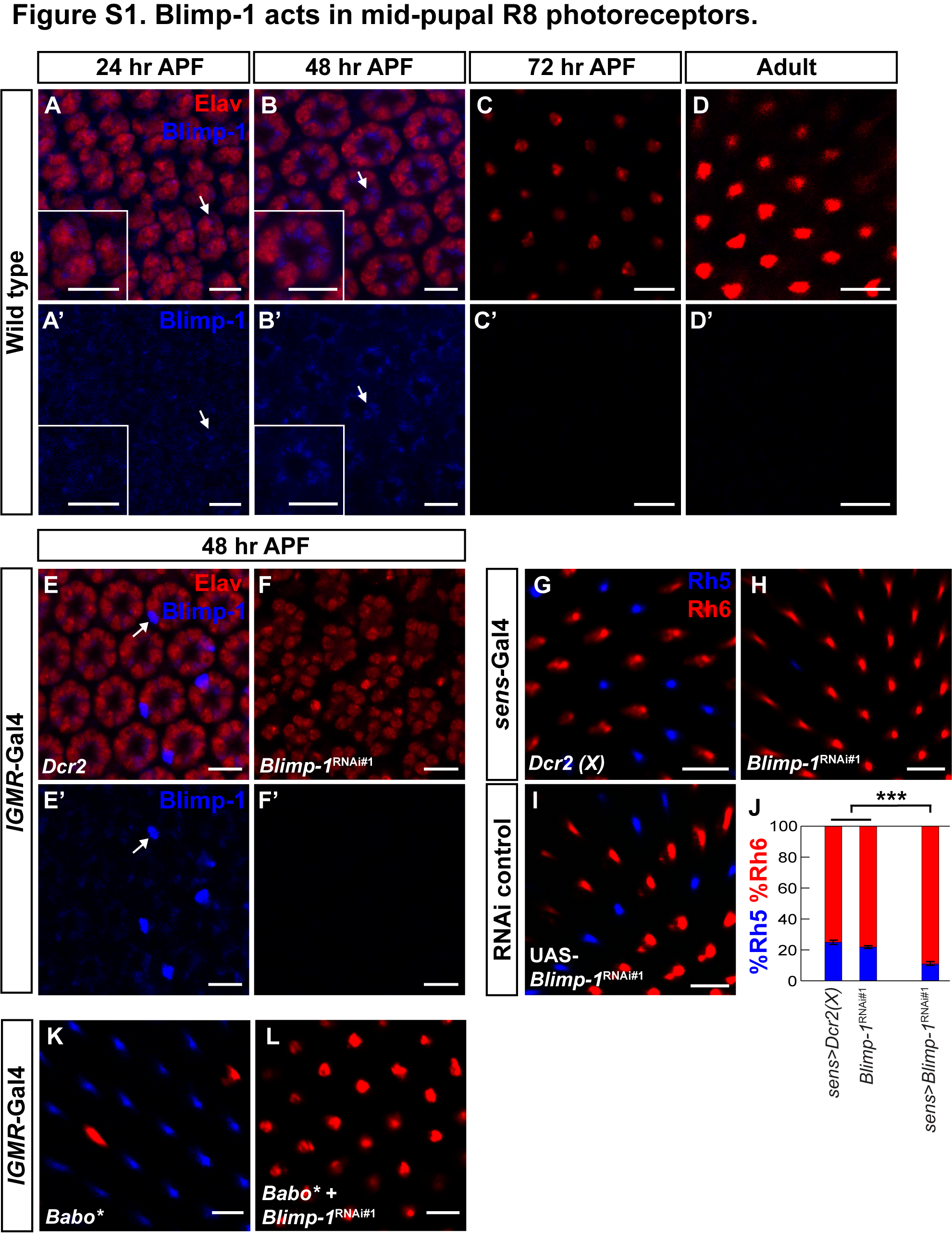

Supplement: Supplementary file 4 [file Image1.TIF]

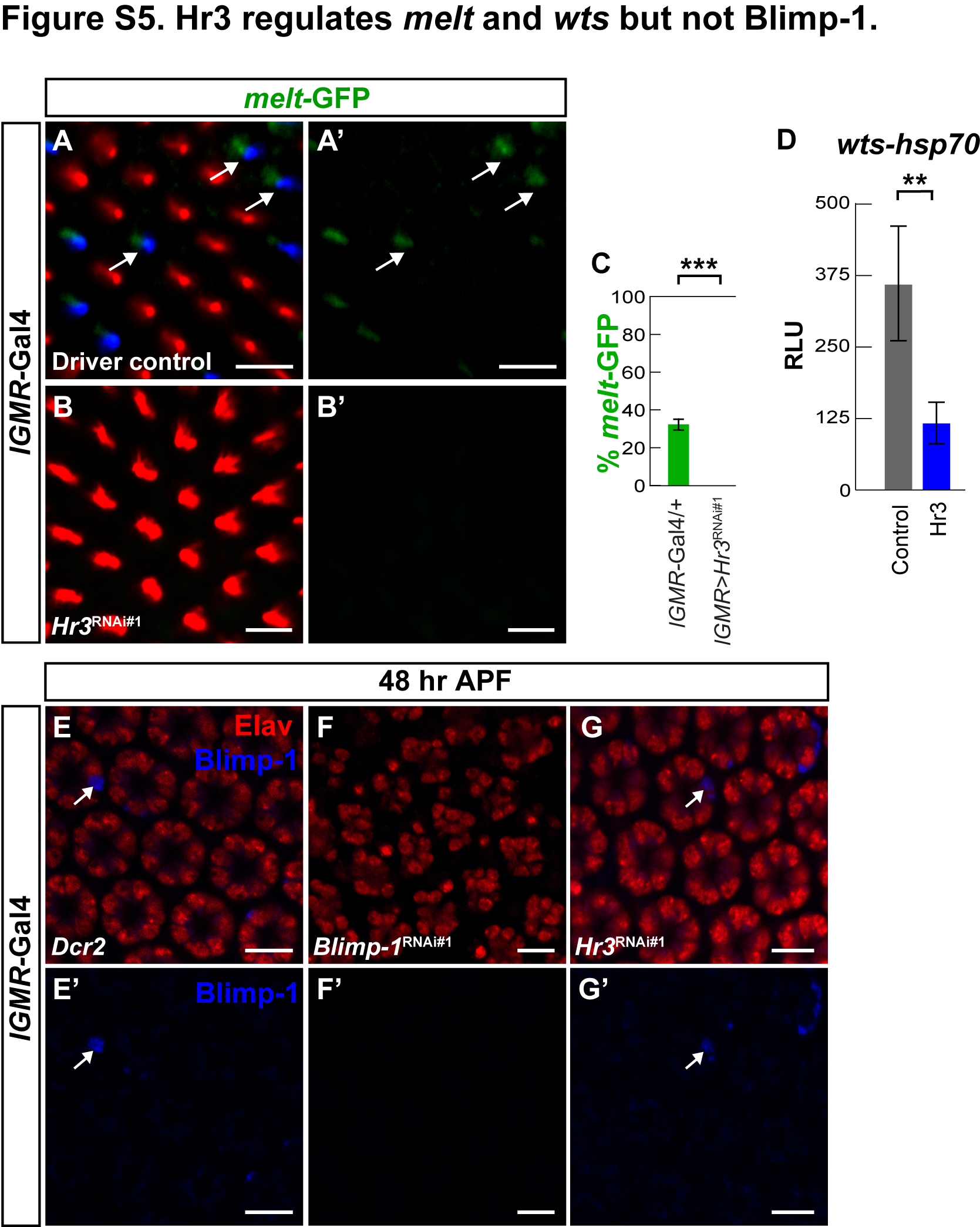

Supplement: Supplementary file 5 [file Image5.TIF]
